# A semi-supervised approach for predicting cell-type specific functional consequences of non-coding variation using MPRAs

**DOI:** 10.1038/s41467-018-07349-w

**Published:** 2018-12-05

**Authors:** Zihuai He, Linxi Liu, Kai Wang, Iuliana Ionita-Laza

**Affiliations:** 1 Department of Biostatistics, Columbia University, New York, 10032 NY USA; 2 Department of Statistics, Columbia University, New York, 10027 NY USA; 3 Raymond G. Perelman Center for Cellular and Molecular Therapeutics, Children’s Hospital of Philadelphia, Philadelphia, PA 19104 USA

## Abstract

Predicting the functional consequences of genetic variants in non-coding regions is a challenging problem. We propose here a semi-supervised approach, GenoNet, to jointly utilize experimentally confirmed regulatory variants (labeled variants), millions of unlabeled variants genome-wide, and more than a thousand cell/tissue type specific epigenetic annotations to predict functional consequences of non-coding variants. Through the application to several experimental datasets, we demonstrate that the proposed method significantly improves prediction accuracy compared to existing functional prediction methods at the tissue/cell type level, but especially so at the organism level. Importantly, we illustrate how the GenoNet scores can help in fine-mapping at GWAS loci, and in the discovery of disease associated genes in sequencing studies. As more comprehensive lists of experimentally validated variants become available over the next few years, semi-supervised methods like GenoNet can be used to provide increasingly accurate functional predictions for variants genome-wide and across a variety of cell/tissue types.

## Abstract (web summary)

Predicting the functional consequences of non-coding genetic variants is a challenge. Here, He et al. present GenoNet, a semi-supervised method that combines information from experimentally confirmed regulatory variants with cell type- and tissue specific annotation for function prediction.

## Introduction

Determining the functional consequences of genetic variants is a difficult problem in human genetics. Our understanding of the genetic code and splicing enables us to identify variants that are likely functional in protein-coding regions, but accurately predicting the functional effects of variants in non-coding regions is much more difficult^1^. Multiple lines of evidence support an important functional role for variants in non-coding regions. For example, comparative genomic studies show that most of the mammalian conserved and recently adapted regions reside in the non-coding part of the genome. In addition, genome-wide association studies (GWAS) have identified a large number of non-coding variants that are likely to be involved in both genetic and epigenetic gene regulation in a highly context-specific manner^2^. Therefore, accurately predicting both organism level and cell type/tissue-specific functional consequences of non-coding variation is of great interest.

There are several possible approaches to predict the functional effects of genetic variants^3^. In the experimental approach (e.g. massively parallel reporter assays (MPRAs), CRISPR/Cas9-mediated in situ saturating mutagenesis), the functional effect of a variant is measured by evaluating the phenotypic consequence of the corresponding sequence alteration (e.g. by measuring the impact of individual alleles on gene expression in a particular context)^4–6^. This is considered the gold-standard approach, but it is quite laborious to perform in a comprehensive manner for large sets of genetic variants. More often, functional effects are derived using alternative approaches. One commonly used method is based on an evolutionary perspective, whereby functional effects are assessed by the extent of evolutionary conservation at the position of interest. The classical evolutionary approach relies on accurate multispecies alignment, which makes it challenging to identify certain functional elements, such as elements constrained only within the human species, although several methods have been recently proposed to identify primate- or human-specific conserved elements^7–9^. Evolutionary approaches also pose an additional challenge, namely they cannot reveal the relevant cell type or tissue. Another popular approach is the biochemical approach, based on ChIP and/or DNase I hypersensitivity assays, with the caveat that such biochemical signatures can occur stochastically, and hence do not completely imply functionality. Therefore, depending on the approach, functional effect can have different meanings in different contexts. This creates challenges for meaningful comparisons among the different approaches.

The rapid development of massively parallel sequencing technologies has made possible large-scale epigenetic projects such as the Encyclopedia of DNA Elements (ENCODE), Roadmap Epigenomics, and BLUEPRINT^10–12^. These projects make available various epigenetic features, including histone modifications and chromatin accessibility, genome-wide in over a hundred different tissues and cell types. Over the past few years, several computational approaches have been proposed to integrate these epigenetic features to predict the organism level and cell type/tissue-specific functional consequences of genetic variants^13–20^. Many of these methods are unsupervised due to the relative scarcity of high-quality labeled data that could be used in supervised approaches. While the unsupervised approaches can be advantageous when the amount and quality of labeled data are limited, supervised methods that make use of high-quality labeled data are able to adaptively learn which functional annotations will help to better discriminate between functional and non-functional variants. Recent developments in high-throughput assays to assess the functional impact of variants in regulatory regions (e.g. MPRAs, CRISPR/Cas9-mediated in situ saturating mutagenesis) can lead to the generation of high-quality data on the functional effects of genetic variants in various contexts. Although these experimental approaches are currently quite laborious and difficult to implement, data on even modest number of variants from such experiments can be used to train (semi-)supervised approaches for improved prediction accuracy.

**Fig. 1:**
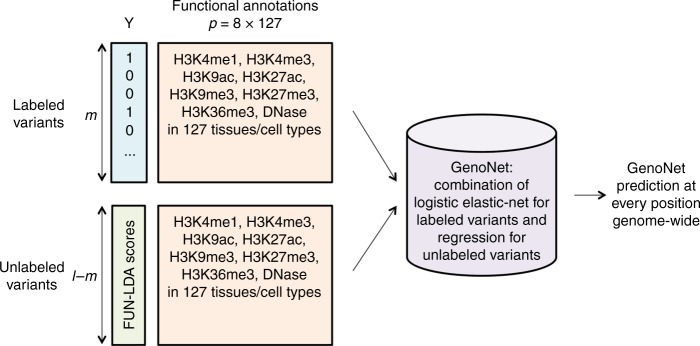
GenoNet workflow. GenoNet integrates experimentally validated variants (${{m}} \ge 0$ labeled variants), millions of unlabeled variants genome-wide (***l***–***m*** unlabeled variants), and more than a thousand cell type/tissue-specific functional annotations (*p*) on each variant to predict the functional consequences of genetic variants at positions genome-wide

Here we propose a semi-supervised method, GenoNet, paired with efficient computational techniques for functional prediction at organism and/or cell type/tissue level. As a semi-supervised method, GenoNet can jointly use labeled data (several dozens to thousands of experimentally confirmed labeled variants) and unlabeled data (millions of unlabeled variants across the genome) for improved prediction accuracy (Fig. 1), and that is not possible for existing functional prediction methods that fall in either the supervised or the unsupervised class. We applied GenoNet to predict the functional effects of non-coding variants, taking advantage of available functional labels from MPRAs, and comprehensive tissue-specific epigenetic features. Through comparisons using several experimentally derived datasets, we show that the proposed method achieves substantially better prediction accuracy over the existing methods, especially so at the organism level. We show how GenoNet can be used to aid in the fine mapping at GWAS loci, as well as in the discovery of disease-associated genes. These applications clearly show the importance of tissue/cell type-specific scores in gene discovery for complex traits.

## Results

### Overview of the method

We propose a semi-supervised regularization algorithm, referred to as GenoNet, for functional prediction at cell type/tissue level using labeled data from MPRA experiments (when available) and genome-wide functional annotations in 127 different cell types and tissues from the ENCODE and Roadmap Epigenomics projects. The key idea of this semi-supervised algorithm is to use supervised algorithms (e.g. logistic Elastic-net, LASSO, ridge regression, etc.) on a limited number of labeled variants, and incorporate the rich information on the genome-wide unlabeled variants (marginal distribution of functional annotations) for improved prediction accuracy^21–23^. Let $\hat f$ be the estimated prediction function, then we solve

$$\hat f = \mathop {{{\mathrm{argmin}}}}\limits_f \mathop {\sum }\limits_{i = 1}^m l_{\rm P}\left( {Y_i,f\left( {{\mathbf{X}}_{\mathbf{i}}} \right)} \right) + \gamma _I\mathop {\sum }\limits_{i = 1}^l \left( {\hat Y_i^u - f\left( {{\mathbf{X}}_{\mathbf{i}}} \right)} \right)^2,$$

where $l_{\rm P}$ is the penalized log-likelihood for the labeled data. Here we adopt Elastic-net because of its superior performance when the features are correlated and have sparse non-zero coefficients^21^. We further justify this choice of supervised algorithm for GenoNet via numerical simulations (Methods). $Y_i \in \left\{ {0,1} \right\}$ are the labels for *m* variants with MPRA validated labels; $\hat Y_i^u \in \left[ {0,1} \right]$ are the predicted values for a large number (*l*) of variants from a prior unsupervised method. We adopt FUN-LDA score in the current GenoNet algorithm because it is one of only a handful of tissue-specific functional scores available genome-wide, recognizing that other unsupervised scores can be readily incorporated into GenoNet in the future^18^; **X**_**i**_ are the functional annotations; *γ*_*I*_ is a tuning parameter that controls how the unlabeled data are being used. When *γ*_*I*_ = 0, the method is fully supervised; when *γ*_*I*_ = ∞, the method is fully unsupervised (Supplementary Table 1). The tuning parameter *γ*_*I*_ is chosen to maximize the area under the receiver operating characteristics curve (AUROC) by tenfold cross-validation. The workflow is depicted in Fig. 1. We present the details in the Methods section.

### Cell type/tissue-specific functional prediction

We compared GenoNet scores with five existing scores for cell type/tissue-specific functional effects and seven existing scores for organism-level functional effects. For cell type/tissue-specific prediction: FUN-LDA, GenoSkyline, quantitative DNase, deltaSVM, DeepSEA (tissue-specific version); for organism level: Eigen/Eigen-PC, CADD, DANN, FunSeq2, LINSIGHT, CATO, DeepSEA (functional significance score; brief descriptions of the different methods are given in the Supplemental Material)^13–20^. We compare the methods in terms of AUROC, area under the precision recall curve (AUPR), and Pearson correlation between the predicted scores on test variants and the true labels (COR). All three criteria have been widely used in the literature to measure prediction accuracy, where COR measures how the predicted values are correlated with the true labels, and AUROC/AUPR are based on the ranks of the predicted scores. We expect that a desirable method performs well in terms of all three criteria.

To examine whether the proposed method can predict cell type/tissue-specific regulatory variants, we took advantage of MPRA validated variants in three cell lines (lymphoblastoid—LCLs, liver carcinoma—HepG2, and erythrocytic leukemia—K562 cell lines). We present the details on these MPRA datasets in the Methods section. We used a 4:1 random partition of each dataset to train/test the method (in order to test the method on a dataset independent of training data, and thus avoid over-fitting issues). Specifically, for each replicate, the dataset is evenly divided into five parts: four parts are used as training data and one part as test data. Details on model training can be found in the Methods section. We trained GenoNet with tenfold cross-validation on the training data only, and calculated functional predictions on the independent test data. We calculate the AUROC, AUPR, and COR based on the average prediction for each variant when it is in the test data using 1000 replicates of the 4:1 random partition to reduce the variation due to resampling.

**Fig. 2:**
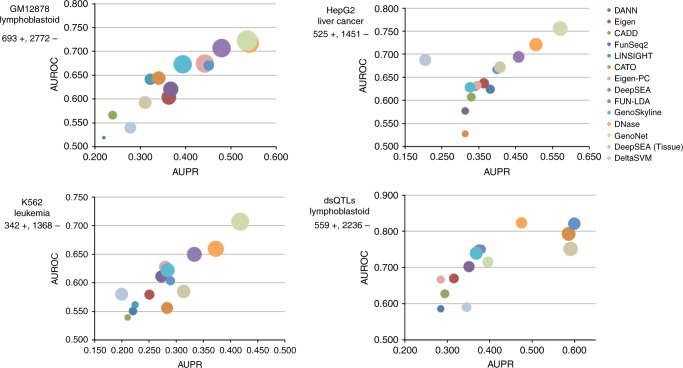
Cell type/tissue-specific prediction of regulatory variants. Each figure presents the AUPR (area under the precision recall curve), AUROC (area under the receiver operating characteristics curve), and COR (Pearson correlation between predicted and true labels). *X* axis presents AUPR; *Y* axis presents AUROC; the bubble size represents COR. GM12878: MPRA validated variants in lymphoblastoid cells (693 positive variants, 2772 control variants). HepG2: MPRA validated variants in liver carcinoma cells (525 positive variants, 1451 control variants). K562: MPRA validated variants in erythrocytic leukemia cells (342 positive variants, 1368 control variants). dsQTLs: a set of DNase I–sensitivity quantitative trait loci identified in a collection of human lymphoblastoid cell lines (559 positive variants, 2236 control variants). GenoNet: the proposed semi-supervised algorithm

We compare the proposed methods with existing tissue-specific functional scores including GenoSkyline, FUN-LDA, DNase, deltaSVM, and DeepSEA (tissue-specific version) and present the results in Fig. 2. (The precise AUROC/AUPR/COR values are summarized in Supplementary Table 2.) We also evaluate the performance of existing organismal functional scores Eigen/Eigen-PC, CADD, DANN, FunSeq2, LINSIGHT, CATO, and DeepSEA on these datasets. We observed that GenoNet exhibits the largest AUROC/COR (LCLs: 0.723/0.442; HepG2: 0.756/0.429; K562: 0.707/0.341) compared to existing functional scores. It also has the largest AUPR for HepG2 (0.571) and K562 (0.418), and the second largest AUPR for LCLs (0.536; the largest AUPR is 0.540 for DNase). These results show that a semi-supervised method like GenoNet, which adaptively makes use of both labeled data (experimentally confirmed regulatory variants) and unlabeled data, outperforms the existing unsupervised scores in terms of all three criteria, with substantial improvements in AUROC. In addition, we observed that organism-level functional scores do not perform as well as the tissue-specific methods in predicting these tissue-specific functional variants. For example, Eigen has AUROCs of 0.604 for LCLs, 0.637 for HepG2, and 0.579 for K562. Similarly, other organism-level predictions such as LINSIGHT, CATO, and DeepSEA exhibit generally lower AUROC, AUPR, and COR in the three cell lines than cell type/tissue-specific predictions.

We additionally evaluate the performance on a collection of DNase I–sensitivity quantitative trait loci (dsQTLs), originally identified using DNase I sequencing data from human LCLs^24^. In Li et al.^25^, the authors selected a subset of dsQTLs with association *p*-value <1 × 10^−5^, and residing within 100-bp of the corresponding DNase I–hypersensitive sites, resulting in a final set of 559 dsQTLs. Then they randomly selected as controls 2236 common single nucleotide polymorphisms (SNPs) (minor allele frequency >5%) from the top 5% of DNase I sensitivity sites used in the original study. We observed that deltaSVM, DeepSEA (the functional significance score), CATO, and DNase have better performance than other methods on this dsQTL dataset (e.g. deltaSVM AUC = 0.751 vs. GenoNet AUC = 0.716), although they have lower accuracy on the previously discussed MPRA validated datasets (e.g. for GM12878, deltaSVM AUC = 0.593 vs. GenoNet AUC = 0.723). Since most dsQTLs are located near the target DNase I hypersensitive sites, we expect that methods such as DeepSEA, DNase, CATO, and deltaSVM perform well on these datasets. In addition, we observed that the DeepSEA tissue-specific score does not perform as well as the organism-level DeepSEA score (the functional significance score).

### Organism-level functional prediction

The cell type/tissue-specific functional predictions for the tissues/cell types in ENCODE and Roadmap Epigenomics provide a comprehensive profile of the functional consequences of a variant across various tissues. However, many available functional prediction scores are available only at the organism level. We show that the organism-level predictions based on the tissue-level predictions (i.e. maximum of cell type/tissue-specific GenoNet scores) are more accurate than existing predictions at the organism level using several different datasets with different ways to define “functional” variants.

**Fig. 3:**
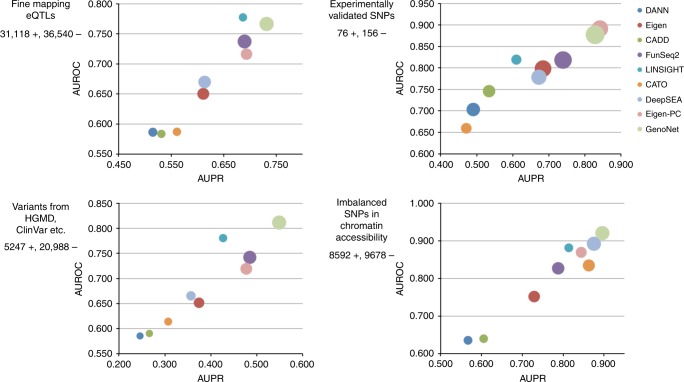
Organismal-level prediction of regulatory variants. Each figure presents the AUPR (area under the precision recal curve), AUROC (area under the receiver operating characteristics curve), and COR (Pearson correlation between predicted and true labels) for different organism-level prediction methods. *X* axis presents AUPR; *Y* axis presents AUROC; the bubble size presents COR. Fine mapping eQTLs: Uniformly processed fine-mapping eQTLs from 11 studies (31,118 positive variants, 36,540 control variants); Experimentally validated SNPs: manually curated experimentally validated regulatory SNPs (76 positive variants, 156 control variants); Refined causal SNPs: refined causal SNPs in the non-coding regions from different resources including HGMD, ClinVar, OregAnno, and variants from fine-mapping candidate causal SNPs for 39 immune and non-immune diseases (5247 positive variants, 20,988 control variants); Allelic imbalanced SNPs: allelic imbalanced SNPs in chromatin accessibility using a large number of DNase-seq assays (8592 positive variants, 9678 control variants)

First, we generate GenoNet predictions for a collection of expression quantitative trait loci (eQTLs) from 11 studies on seven tissues/cell lines^26^. Li et al. restricted to the most highly associated eQTLs for each tissue/cell type, for a total of 31,118 eQTLs^25^. They also selected 36,540 background SNPs near the transcription start sites (TSS) of randomly selected genes as control variants. We present the results in Fig. 3. (The precise AUROC/AUPR/COR values are summarized in Supplementary Table 3.) We observed that GenoNet has larger AUPR and COR (0.731/0.369) than other methods, with the second largest AUPR and COR being 0.693 for Eigen-PC and 0.351 for FunSeq2, respectively. It also has the second largest AUROC (0.766), which is comparable to the largest AUROC (0.778) from LINSIGHT.

Next, we generate GenoNet predictions for three collections of variants, including (1) 76 experimentally validated regulatory SNPs; (2) 5247 refined causal SNPs in non-coding regions from HGMD, ClinVar, and OregAnno; (3) variants identified by a recent fine-mapping study for 39 immune and non-immune diseases^25,27^. For the experimentally validated regulatory SNPs, we select 156 frequency-matched background variants within 10 kb as controls, same as Li et al.^25^. For the refined causal SNPs, we paired each positive variant with four randomly selected frequency-matched non-coding SNPs outside of the refined causal single nucleotide polymorphisms (SNPs). We present the results in Fig. 3. As shown, GenoNet has the largest AUPR, AUROC, and COR for both datasets, with substantial improvements over competing methods. For the experimentally validated SNPs, Eigen-PC exhibits the largest AUPR/AUROC (0.842/0.892) and second largest COR (0.497), while GenoNet exhibits the second largest AUPR/AUROC (0.828/0.877) and largest COR (0.635). For the refined causal SNPs, the AUPR/AUROC/COR of GenoNet are 0.549/0.811/0.458, while the second largest values are 0.485 (FunSeq2)/0.781 (LINSIGHT)/0.397 (FunSeq2).

Finally, we generated GenoNet predictions for 8592 allelic imbalanced SNPs in chromatin accessibility, previously identified by a large number of DNase-seq assays^28^. We pair them with 9678 controls, which are frequency-matched background SNPs around nearest TSS of randomly selected genes. We present the results in Fig. 3. We observed that GenoNet has the largest AUPR (0.896), AUROC (0.921) and second largest COR (0.612), with the second largest AUPR and AUROC of 0.875 (DeepSEA), 0.892 (DeepSEA), and largest COR of 0.615 (DeepSEA), respectively.

In addition to the AUROC/AUPR/COR comparisons detailed above, the full receiver operating characteristics curves and precision recall curves can be found in Supplementary Figures 1 and 2. Overall, we observed that the AUROC and AUPR curves of GenoNet are generally above the curves for other methods, and GenoNet performs particularly well in the regions with lower recall (or higher precision) in the AUPR curves.

### Transferability of cell type/tissue-specific predictions

We additionally evaluated the transferability of cell type/tissue-specific predictions of regulatory variants across tissues and present the results in Table 1. We observed that GenoNet scores trained in one cell line exhibit substantially lower AUROC in predicting MPRA validated variants in another cell line. For example, GenoNet trained using variants validated in LCLs has AUROC 0.627 for variants in liver carcinoma (HepG2) and 0.604 for variants in erythrocytic leukemia (K562), much lower than the AUROC of 0.723 for LCLs. Similar results hold for GenoNet trained using MPRA validated variants in the other two cell lines. We note that the training data and test data are independent even for the same cell line because of the 4:1 random partition design, which eliminates the possibility of inflated AUROC values due to over-fitting. These results demonstrate that GenoNet can accurately distinguish regulatory variants in one tissue vs. another tissue.

**Table 1:** Transferability of cell type/tissue-specific predictions of regulatory variants across tissues

	GM12878 (lymphoblastoid cells)			HepG2 (liver carcinoma cells)			K562 (erythrocytic leukemia cells)		
Training set	AUPR	AUROC	COR	AUPR	AUROC	COR	AUPR	AUROC	COR
GM12878	**0.536**	**0.723**	**0.442**	0.400	0.627	0.212	0.322	0.604	0.183
HepG2	0.313	0.536	0.123	**0.571**	**0.756**	**0.429**	0.277	0.592	0.125
K562	0.327	0.530	0.150	0.348	0.567	0.147	**0.418**	**0.707**	**0.341**

For each row, GenoNet was trained using labels from different tissues. Each cell presents the AUPR (area under the precision recall curve), AUROC (area under the receiver operating characteristics curve), and COR (Pearson correlation between predicted and true labels) calculated based on the average prediction for each variant when it is in the test data using 1000 replicates. For each replicate, the datasets were evenly divided into five parts: four as training data, and one as test data. GM12878: MPRA validated variants in lymphoblastoid cells (693 positive variants, 2772 control variants). HepG2: MPRA validated variants in liver carcinoma cells (525 positive variants, 1451 control variants). K562: MPRA validated variants in erythrocytic leukemia cells (342 positive variants, 1368 control variants). The highest value in each column is bolded

**Fig. 4:**
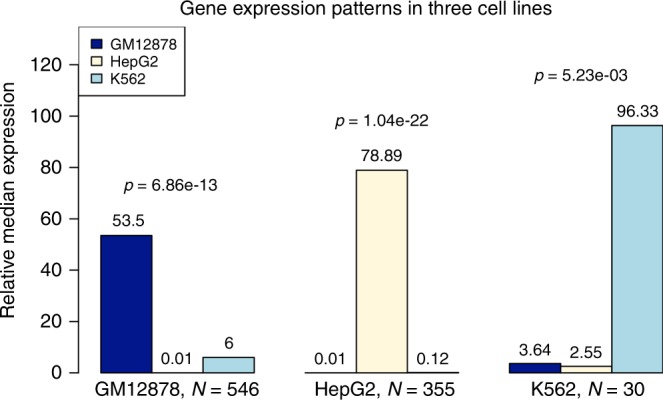
Gene expression patterns for genes with cell-line-specific functional variants in promoters. Each bar presents the median expression in one cell line for genes with cell-line-specific functional variants in promoters relative to the median expression for these genes in the other two cell lines combined. The *p*-values are calculated by testing whether the expression in the matched cell line is greater than the other two cell lines combined, using the Wilcoxon rank sum test. The GenoNet threshold to define functional/non-functional variants in promoter region of a gene is 0.9/0.5

To further investigate this lack of transferability of predictions across tissues, we evaluated whether tissue-specific functional elements predicted by GenoNet correlate with known biological knowledge of the tissues or cells. To test this, we selected three ENCODE cell lines (LCLs, HepG2, K562) and then defined sets of genes for each cell line, e.g. for GM12878 (LCLs), consisting of genes whose promoter regions (1 kb from the TSS) contain at least one variant with GenoNet score >0.9 in LCLs and no variant with GenoNet score >0.5 in HepG2 and K562 (we evaluated different thresholds on the GenoNet score and the results remain qualitatively the same; Supplementary Figures 3 and 4). Then we compared the median gene expression in GM12878, HepG2, and K562 for genes in this set. We present the results in Fig. 4. We observed that the median gene expression in GM12878 is significantly higher (53.5-fold, Wilcoxon rank sum test *p* = 6.86 × 10^−13^) than in the other two cell lines combined. We did the same analysis for HepG2 and K562, respectively, and the results consistently show that the genes selected based on cell line-specific GenoNet scores for variants in promoters tend to have higher expression in the matched cell line compared with the other cell lines.

### Enrichment analyses of eQTLs and dsQTLs

Using the MPRA-based GenoNet scores, we have performed additional enrichment analyses using several eQTL and dsQTL datasets to investigate how the GenoNet score is able to distinguish eQTLs/dsQTLs (as a surrogate for functional variant) from a background variant in the genome. For eQTLs, we use a collection of 17,530 lead eQTLs (those variants most associated with expression levels) in LCLs from the Geuvadis project and the TwinsUK cohort^29^. Among them, we further considered a subset (3268) of eQTLs with fine mapping probability >0.5, calculated based on a resampling-based approach called CaVEMaN^29^. We also analyzed a collection of 5684 eQTLs unique to LCLs (i.e. only significant in LCLs) from the GTEx project^30^. For dsQTLs, we utilized the aforementioned collection of 559 dsQTLs in human LCLs, originally identified using DNase I sequencing data^24^. In addition, we considered separately 102 dsQTLs that are also associated with variation in expression levels of nearby genes (eQTLs)^24^.

**Fig. 5:**
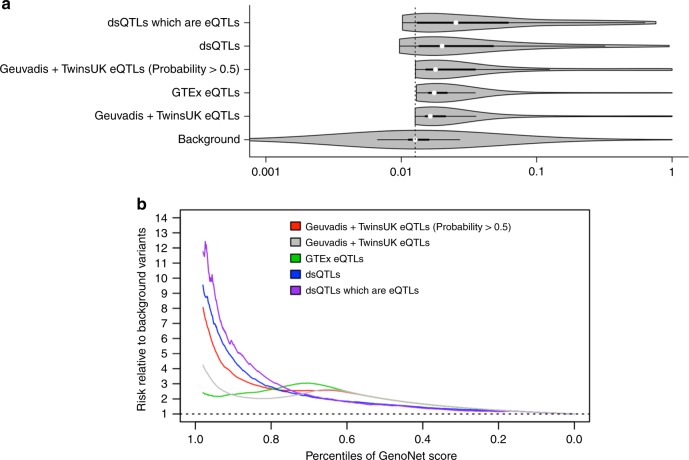
Enrichment analyses using eQTLs/dsQTLs. **a** Violin plots comparing the distributions of GenoNet scores for different sets of eQTLs or dsQTLs identified in previous studies. **b** Each curve represents the probability of a variant being eQTL or dsQTL, conditional on its GenoNet score being greater than a given percentile, relative to randomly selected background variants. We note that because only a small subset of eQTLs have high GenoNet scores in these datasets, the estimation of the relative risk in the tails can be unstable

We first present the distributions of GenoNet scores for variants in the different sets of eQTLs and dsQTLs described above in Fig. 5a, and compare with the distribution of scores for 2 million background variants across the genome. We observed that eQTLs with fine mapping probability >0.5 tend to have significantly higher GenoNet scores than eQTLs in general, as expected. We also observed that dsQTLs tend to have higher GenoNet scores than eQTLs, likely due to the higher chance for dsQTLs near the DNase I-hypersensitive sites to be functional relative to eQTLs, and concordant with the hypothesis that changes in chromatin accessibility may be an important mechanism by which genetic variants affect gene expression. Overall, eQTLs and dsQTLs tend to have significantly higher GenoNet scores than background variants (*p* < 10^−20^).

We then estimated the probability of a variant to be an eQTL or dsQTL conditional on its GenoNet score being greater than a given percentile, relative to a randomly selected background variant, e.g. $r = \frac{{P\left( {{\mathrm{variant}}\,{\mathrm{is}}\,{\mathrm{eQTL|GenoNet}} > q_x} \right)}}{{P\left( {{\mathrm{variant}}\,{\mathrm{is}}\,{\mathrm{eQTL}}} \right)}}$, where *q*_*x*_ is the *x*th percentile of GenoNet score (Fig. 5b). Compared to a background variant, we observed that a variant with GenoNet score greater than the 98th percentile is 12 times more likely to be both a dsQTL and an eQTL, nine times more likely to be a dsQTL, eight times more likely to be a fine-mapped eQTL (probability > 0.5), and two to four times more likely to be eQTL.

### Fine-mapping of *MIR137* schizophrenia risk locus

The functional predictions from GenoNet can also be used to help in fine-mapping at previously identified GWAS loci. To illustrate GenoNet’s potential in this setting, we show the case of a leading non-coding GWAS locus in schizophrenia, namely the *MIR137* locus^31^. In Forrest et al., the authors focus on the *MIR137* locus, and using open chromatin analyses in hiPSC-differentiated neurons they prioritize one SNP, rs1198588, among 23 equally associated SNPs at this locus^32^. Excitatory neurons derived from hiPSCs with CRISPR/Cas9-edited rs1198588 showed changes in *MIR137* gene expression and impaired neurodevelopment. An additional functional rare variant known to increase the risk to schizophrenia (1:g.98515539 A > T) is also within a neuronal open chromatin region proximal to the *MIR137HG* TSS^33^.

**Fig. 6:**
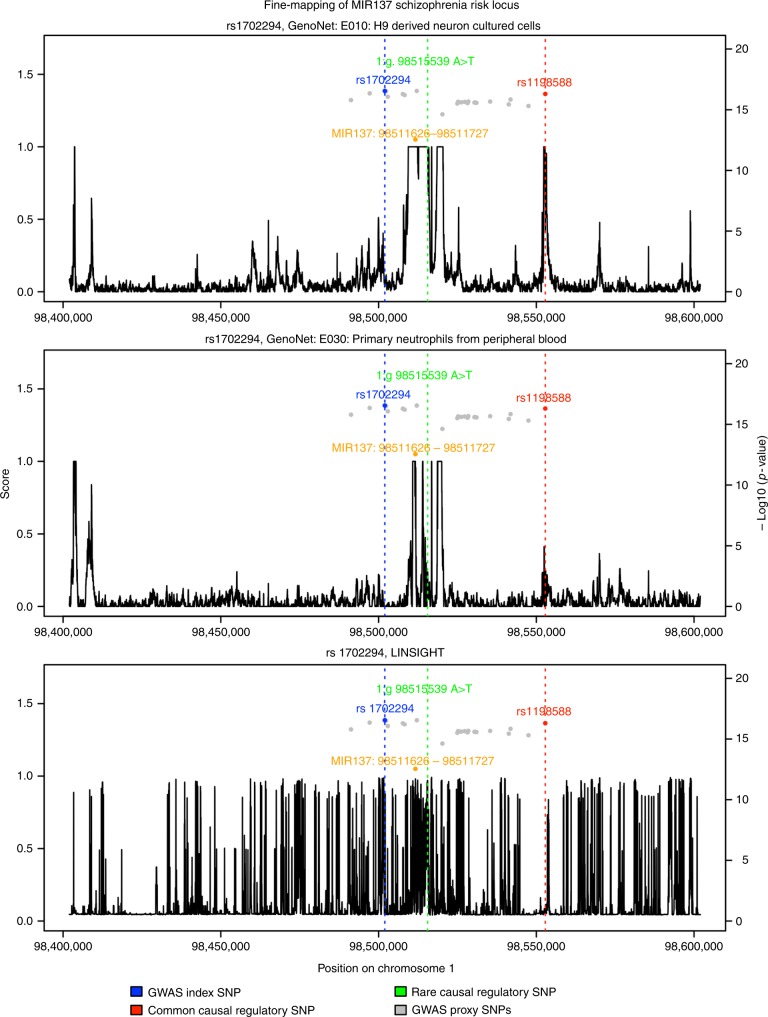
Fine-mapping of MIR137 schizophrenia risk locus. The plots present GenoNet predictions in a 200-Kb region centered at the GWAS index SNP at this locus, rs1702294, in H9-derived neuronal cultured cells as well as in primary neutrophils from peripheral blood. In addition, we show predictions in this region from LINSIGHT, an organism-level functional prediction approach. 1:g.98515539 A > T is a known rare causal variant in schizophrenia

We sought to investigate the use of our GenoNet predictions in prioritizing these two known causal regulatory SNPs at the *MIR137* locus. In Fig. 6, we show GenoNet predictions in H9-derived neuronal cultured cells in a 200-Kb region centered at the GWAS index SNP at this locus, rs1702294. In addition, we show GenoNet predictions for primary neutrophils from peripheral blood, along with predictions from LINSIGHT, a recently developed organism-level functional prediction approach. As shown, GenoNet is successful in predicting as functional the CRISPR/Cas-9 validated SNP, rs1198588 (GenoNet score = 0.656), as well as the location of the known functional rare risk variant, 1:g.98515539 A > T (GenoNet score = 1), in H9-derived neuronal cultured cells, while in the case of primary neutrophils from peripheral blood, GenoNet scores are low (GenoNet score = 0.012 for rs1198588; 0.222 for 1:g.98515539 A > T). Compared with GenoNet, organism-level functional prediction method LINSIGHT performs much worse and does not accurately predict the locations of the two known functional variants at this locus. In Supplementary Figure 5, we show further comparisons with additional functional scores, including Eigen, Eigen-PC, FUN-LDA, and individual epigenetic annotations such as DNase, H3K27ac, H3K9ac, H3K4me1, and H3K4me3. As shown, FUN-LDA performs quite well in this setting, similar to GenoNet. Eigen, like LINSIGHT, is not able to achieve accurate predictions, while Eigen-PC performs better, assigning a high functional score for the common causal SNP, rs1198588. Of the individual epigenetic scores in H9-derived neuronal cultured cells (DNase, H3K27ac, H3K9ac, H3K4me1, H3K4me3), only H3K27ac assigns a high score to the common causal regulatory SNP, although it misses the rare functional variant. Overall, GenoNet and FUN-LDA, as integrative methods, perform best in predicting as functional the two known regulatory variants at the *MIR137* locus. As an additional analysis, we have checked whether the CRISPR/Cas-9 validated SNP falls into a transcription factor binding site, by checking the TFBS identified by ChIP-seq experiments in ENCODE for the MIR137 locus. As shown in Supplementary Figure 6, there are several TFBS at the MIR137 locus, but they are not very close to the CRISPR/Cas-9 validated SNP.

### Integrative analysis of rare variants in Metabochip regions

The functional predictions made by GenoNet not only help with variant interpretation and fine-mapping of GWAS loci, but also benefit the discovery of new disease-associated genes. Gene-based tests for sequencing data such as Burden and SKAT have shown limited success in empirical studies so far, likely a result of modest sample sizes for sequence-based studies performed, as well as sub-optimal implementations of such tests^34^. It has been recently shown how incorporating functional predictions in sequence-based association tests can lead to improved power of gene-based association tests^35^. The functional predictions we provide in 127 different cell types/tissues provide comprehensive profiles across large number of tissues/cell types, which can be integrated with sequence-based data.

We incorporated the GenoNet scores into an integrative test (referred to as FST-GenoNet, FST-functional score test), and applied it to a meta-analysis of rare variants (minor allele frequency <0.05) in Metabochip data on 12,281 individuals from eight studies for lipid traits (total cholesterol (CHOL), high-density lipoproteins cholesterol (HDL), low-density lipoproteins cholesterol (LDL), and triglycerides (TG))^35–37^. The eight studies include Finnish: FUSION stage 2 (2741), D2D 2007 (2108), DPS (429), METSIM (1439), DR’s EXTRA (1242); Norwegian: HUNT, Tromso (2793 for the two studies together); German: DIAGEN (1529). We adjusted for gender, age, age squared, and type 2 diabetes status for each individual study. We analyzed the two Norwegian cohorts jointly, with an additional covariate for study of origin. Birthplace was additionally adjusted for FUSION stage 2. We did not adjust for gender for METSIM because it only contains males. Samples and SNPs with call rates <98% are excluded from the analysis. We also exclude samples when the outcomes are missing for each trait. We applied normal quantile transformation to each trait and evaluated 266 genes located in the 99 gold fine-mapping regions, meta-analyzing the summary statistics from the eight individual studies. We compared the results of FST-GenoNet with the original test without integrating any functional information (referred to as FST-O, equivalent to SKAT-O) and a test integrating organism-level predictions FST-LINSIGHT (FST integrating the functional score LINSIGHT), and defined the significant genes those with *p*-values <2.5 × 10^−6^.

**Fig. 7:**
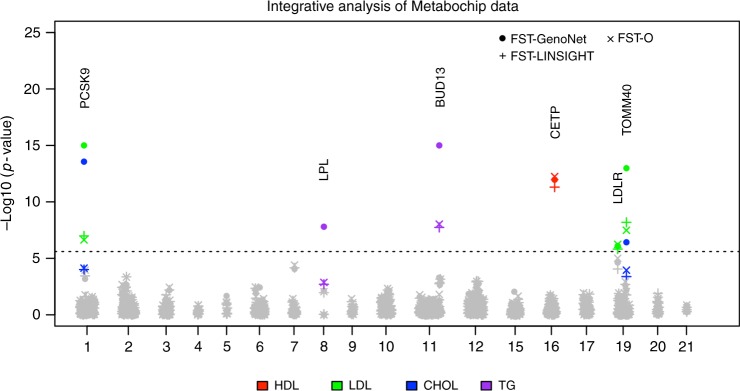
Application of GenoNet scores to the meta-analysis of rare variants in Metabochip data. The figure shows the *p*-values of 266 genes in the 99 gold fine-mapped regions in the Metabochip study. FST-GenoNet: the functional score test proposed by He et al.^35^, integrating the GenoNet scores over 127 Roadmap tissues. FST-O: the original test combining burden and dispersion tests without integrating any functional scores (equivalent to SKAT-O); FST-LINSIGHT: the FST test integrating the organism-level prediction method LINSIGHT. The dashed line corresponds to the gene-based genome-wide significance level (2.5 × 10^−6^). When either FST-GenoNet, FST-O, or FST-LINSIGHT passes the significance level, all methods are highlighted for that gene-trait association. The *X* axis presents the chromosome on which the genes reside

We summarize the significant genes identified by either FST-GenoNet, FST-O, or FST-LINSIGHT in Fig. 7. We observed that by incorporating GenoNet scores, we are able, for some genes, to obtain substantially more significant results than those provided by FST-O (e.g. *TOMM40*: 2.4 × 10^−13^ vs. 5.3 × 10^−8^ for LDL, 4.1 × 10^−6^ vs. 3.7 × 10^−4^ for CHOL; *PCSK9*: 1 × 10^−15^ vs. 9.0 × 10^−8^ LDL, 1.1 × 10^−14^ vs. 3.5 × 10^−5^ for CHOL; *BUD13*: 1 × 10^−15^ vs. 1.4 × 10^−8^ for TG; *LPL*: 3.7 × 10^−8^ vs. 8.7 × 10^−4^ for TG), while for *CETP* and *LDLR* the *p*-values from FST-GenoNet and FST-O are of the same order (*CETP*: 1.3 × 10^−13^ vs. 7.5 × 10^−14^ for HDL; *LDLR*: 2.2 × 10^−6^ vs. 9.2 × 10^−7^), while FST-LINSIGHT exhibits very similar *p*-values to FST-O. Among them, *PCSK9*, *LPL, CETP,* and *LDLR* were also reported to be associated with the same traits in both of the two recent meta-analyses of exome-wide association studies of 47,532 East Asian individuals and >300,000 European samples, respectively^38,39^. The QQ plots for these meta-analyses are included in Supplementary Figure 7. We note that genes on the Metabochip sit in previously identified loci, therefore the distribution of *p*-values tends to deviate slightly from the null expectation. Overall the *p*-values from FST-GenoNet are consistent with those from FST-O (equivalent to SKAT-O) and FST-LINSIGHT except in the tails, demonstrating that the type I error rate is well controlled after integrating 127 GenoNet scores. Comparisons with other cell type/tissue-specific tests, such as GenoSkyline and quantitative DNase, are shown in the Supplementary Figure 8. Overall, the results of incorporating these alternative tissue-specific scores are similar to those including GenoNet.

## Discussion

We proposed here a statistical learning framework, GenoNet, to make accurate predictions of both organism-level and cell type/tissue-specific functional consequences of non-coding variation. GenoNet distinguishes itself from existing methods by being semi-supervised, and therefore can jointly use data from several dozens to thousands of experimentally confirmed labeled variants and millions of unlabeled variants across the genome for improved prediction accuracy. The ability to infer cell-type-specific functional elements that regulate target genes will greatly enhance our understanding of what genes contribute to cellular identity and cell lineage differentiation, and better prioritize non-coding variants from GWAS or NGS in the contexts of the cell types that are relevant to the disease under study. It also allows the design of functional genomics experiments that target specific subsets of non-coding regions based on the cell type under study.

The analyses of datasets containing variants with prior experimental or statistical evidence of functional effects (such as MPRA validated variants, eQTLs, dsQTLs, etc.) illustrate that GenoNet has superior prediction accuracy over existing methods. It is worth noting that commonly used organism-level prediction methods such as CADD, DANN, Eigen/Eigen-PC, CATO, DeepSEA, FunSeq2, and LINSIGHT have substantially lower accuracy than GenoNet in predicting functional effects in the example datasets considered here. Furthermore, GenoNet provides a much more detailed overview of the functional effects of genetic variants across different tissues/cell types.

We further demonstrate the use of GenoNet in important applications for complex traits. In particular, we illustrate its ability to pinpoint likely functional variants at GWAS loci using one of the leading loci in schizophrenia, the *MIR137* locus. Compared to organismal-level methods such as LINSIGHT, the GenoNet score is much more effective and correctly predicts the location of two regulatory variants at this locus. The application to the Metabochip data illustrates the advantage of integrating diverse functional scores into gene-based analyses for improved power over commonly used gene-based tests. Given the increasing number of studies generating whole-genome sequencing data for various complex diseases, such integrative analyses for region/gene-based tests can potentially improve the identification of novel regions. A further application to variants involved in 3C chromatin interactions is shown in the Supplemental Materials (Supplementary Table 4, Supplementary Figures 10 and 11)^40^.

We have shown that both the number of labeled variants and the accuracy of the labels are important factors that can affect the accuracy of the semi-supervised approach, although to a lesser extent than that of a purely supervised method (Methods section). More generally, the precise definition of function is important for (semi-)supervised methods. In Supplementary Table 5, we compared the performance of GenoNet scores trained using different definitions of function for labeling functional variants. We show, as known already, that the definition of function plays an essential role for training, and that the different ways to define function lead to different algorithms behaving differently depending on the test datasets. For example, the model trained using eQTLs accurately predicts eQTLs in various test datasets, but performs poorly in predicting dsQTLs and MPRA validated variants (Supplementary Table 5). Overall, GenoNet trained using MPRA regulatory variants has relatively robust predictions for eQTLs and dsQTLs in terms of the AUROC loss, relative to when using the same type of labels (eQTLs or dsQTLs) for training (Supplementary Table 5).

GenoNet is a flexible approach that can incorporate a large number of different types of features. In the present implementation, we have not included transcription factor binding site (TFBS) information in the prediction model because such data (either from ChIP-seq experiments or purely computational predictions based on position weight matrices) are currently incomplete and of varying degrees of reliability. However, it can be helpful to follow up variants with high GenoNet scores, by checking whether particular variants directly disrupt high confidence binding sites and the corresponding transcription factors (see Supplementary Figure 6 for an example). This type of analysis can help further narrow down the list of possible causal variants and can provide more insight into the possible mechanisms of action.

The GenoNet scores are freely available for over a hundred different cell types/tissues in ENCODE and Roadmap on a genome-wide scale. While GenoNet makes use of experimentally validated variants for training whenever such data are available, GenoNet scores can be computed even for those tissues/cell types that currently lack lists of experimentally confirmed variants; in such cases, the GenoNet scores are linear approximations of the FUN-LDA posterior probabilities. As high-quality labels on the functional effects of genetic variants in various cell types and tissues are expected to become increasingly available in the near future, GenoNet can take advantage of such an information for increasingly accurate functional predictions.

## Methods

### MPRA datasets

The first MPRA dataset contains confirmed regulatory variants in LCLs^4^. The authors (Tewhey et al.) have applied a new version of the MPRA to identify variants that show differential expression between alleles (emVars, expression-modulating variants). In particular, they identified 842 emVars out of 32,373 variants in 3642 eQTLs and control regions in LCLs. After excluding the emVars that cannot be mapped to a genomic location using the Ensemble database, we define the remaining 693 emVars as positive variants. Then we matched each emVar with four controls (2772 variants in total), defined as variants that were tested using MPRA but neither allele showed effects on expression (Bonferroni corrected *p*-value > 0.1).

We considered two other MPRA datasets of regulatory motifs, one in liver carcinoma (HepG2) cell line and one in erythrocytic leukemia (K562) cell line. Kheradpour et al. generated these datasets by measuring the transcriptional levels produced by targeted motif disruptions in 2104 candidate enhancers^5^. They compared the expression values for the sequences with the motif with the values for sequences with scrambled versions of the motif. We defined the variants with *p*-values <0.05 as positive variants, and those with *p*-value >0.1 as controls, and removed those variants whose genomic coordinates we could not resolve. The final datasets consist of 525 positive and 1451 control variants for HepG2, and 342 positive and 1368 control variants for K562.

### GenoNet is a semi-supervised approach

Consider *p* standardized functional annotations of *l* genetic variants ${\mathbf{X}}_{\mathbf{i}} = (X_{i1}, \ldots ,X_{ip})$, $i = 1, \ldots ,l$. Without loss of generality, suppose we know the underlying functional status of the first $0 \le m \le l$ variants, $Y_i \in \left\{ {0,1} \right\}$, $i = 1, \ldots ,m$. Let **X** be the functional annotation matrix for all labeled and unlabeled variants; **X**_(**m**)_ be the functional annotations sub-matrix for the labeled variants only; **Y** be the label vector. Let $\hat f$ be the estimated prediction function, then we solve

$$\hat f = \mathop {{{\mathrm{argmin}}}}\limits_f \mathop {\sum }\limits_{i = 1}^m l_{\rm P}\left( {Y_i,f\left( {{\mathbf{X}}_{\mathbf{i}}} \right)} \right) + \gamma _I\mathop {\sum }\limits_{i = 1}^l \left( {\hat Y_i^u - f\left( {{\mathbf{X}}_{\mathbf{i}}} \right)} \right)^2,$$

where $l_{\rm P}$ is the penalized log-likelihood for the labeled data. We adopt Elastic-net because of its superior performance when the features are correlated and have sparse non-zero coefficients^21^; $Y_i \in \left\{ {0,1} \right\}$ are the labels for *m* variants with MPRA validated labels; ${\hat{ Y}}_{{i}}^{{u}} \in \left[ {0,1} \right]$ are the predicted values for a large number (*l*) of variants from a prior unsupervised method. We choose the FUN-LDA score in the current GenoNet implementation because it is one of only a handful of tissue-specific functional scores available genome-wide, recognizing that other unsupervised scores can be readily incorporated into GenoNet in the future^18^. *γ*_*I*_ is a tuning parameter that controls how the unlabeled data are being used^18^. When *γ*_*I*_ = 0, the method is fully supervised; when *γ*_*I*_ = ∞, the method is fully unsupervised (Supplementary Table 1). As *Y*_*i*_’s often consist of a set of positive variants paired with control variants (a case-control design), but $\hat Y_i^u$’s are continuous and randomly sampled from the genome (a cohort design), it is not trivial to find *f* by directly solving the optimization problem. Instead, we solve two optimization problems for labeled data, and unlabeled data (with *γ*_*I*_ = 0 and *γ*_*I*_ = ∞) separately, and then combine the resulting functions.

For *γ*_*I*_ = 0, we define a logistic function $f_1\left( {{\mathbf{X}}_{\mathbf{i}}} \right) = \frac{{\exp \left( {\beta _0 + {\mathbf{\beta }}^{\mathbf{T}}{\mathbf{X}}_{\mathbf{i}}} \right)}}{{1 + \exp \left( {\beta _0 + {\mathbf{\beta }}^{\mathbf{T}}{\mathbf{X}}_{\mathbf{i}}} \right)}}$ and solve the regularization problem $\widehat {f_1} = \mathop {{{\mathrm{argmin}}}}\limits_{f_1} \mathop {\sum }\limits_{i = 1}^m l_{\rm P}\left( {Y_i,f_1\left( {{\mathbf{X}}_{\mathbf{i}}} \right)} \right)$, where

$\ l_{\rm P}\left( {Y_i,f_1\left( {{\mathbf{X}}_{\mathbf{i}}} \right)} \right) = \frac{1}{m}\left\{ {\log \left( {1 + e^{\beta _0 + {\mathbf{\beta }}^{\mathbf{T}}{\mathbf{X}}_{\mathbf{i}}}} \right) - Y_i\left( {\beta _0 + {\mathbf{\beta }}^{\mathbf{T}}{\mathbf{X}}_{\mathbf{i}}} \right)} \right\} + \frac{\lambda }{m}\left[ {\frac{{\left( {1 - \alpha } \right)}}{2}\parallel{\mathbf{\beta }}\parallel_{l2}^2 + \alpha \parallel{\mathbf{\beta }}\parallel_{l1}} \right]$^22^. The *l*_1_ and *l*_2_ penalties are incorporated because the number of labeled variants is often limited. We set *α* = 0.5 and $\lambda = \hat \sigma \left( Y \right)\sqrt {\frac{{2\log p}}{m}}$ where $\hat \sigma \left( Y \right)$ is the sample standard deviation of *Y*. The choice of *α* and *λ* aims to balance the computational efficiency and prediction accuracy and we discuss the details in the Supplementary Information. The method can be applied to data with imbalanced classes (positive vs. control variants) because the coefficients (except the intercept) estimated assuming prospective and retrospective designs are equivalent for logistic regression^22^. In practice, to predict the probability of a new variant to be functional, we set the prevalence to 2% (estimated by Backenroth et al.) and correct the intercept by $\tilde \beta _0 = \beta _0 - \log \frac{{1 - 0.02}}{{0.02}} \times \frac{\pi }{{1 - \pi }}$, where *π* is the proportion of positive variants in the training data^18^.

For *γ*_*I*_ = ∞, we define $f_2\left( {{\mathbf{X}}_{\mathbf{i}}} \right) = \alpha _0 + \alpha ^T{\mathbf{X}}_{\mathbf{i}}$ and solve the ordinary least-square problem $\widehat {f_2} = \mathop {{{\mathrm{argmin}}}}\limits_{f_2} \mathop {\sum }\limits_{i = 1}^l \left( {\hat Y_i^u - f_2\left( {{\mathbf{X}}_{\mathbf{i}}} \right)} \right)^2$. In practice, we randomly selected 100,000 500-bp segments across the genome where each segment includes 20 variants (25 bp apart), for a total of 2 million background variants as unlabeled data. These 2 million background variants are used to estimate $\hat f_2$. Finally, we define

$$\hat f = \left( {1 - \hat \phi } \right)\hat f_1 + \hat \phi \hat f_2,$$

where the tuning parameter $\hat \phi$ is chosen to maximize the AUROC curve by tenfold cross-validation.

The proposed method is connected to some existing machine learning algorithms. For example, manifold regularization is a semi-supervised method with $\hat f = \mathop {{{\mathrm{argmin}}}}\limits_{f \in {\cal H}_K} \mathop {\sum }\limits_{i = 1}^m \left( {Y_i - f\left( {{\mathbf{X}}_{\mathbf{i}}} \right)} \right)^2 + \gamma _A\parallel f\parallel_K + \gamma _I\mathop {\sum }\limits_{i,j = 1}^l \left( {f\left( {{\mathbf{X}}_{\mathbf{i}}} \right) - f\left( {{\mathbf{X}}_{\mathbf{j}}} \right)} \right)^2W_{ij}$, where *W*_*ij*_ are edge weights in the data adjacency graph^41^. The last penalty term is based on the graph Laplacian. GenoNet considers a logistic model for binary labels and replaces the last term by $\mathop {\sum }\limits_{i = 1}^l \left( {\hat Y_i^u - f\left( {{\mathbf{X}}_{\mathbf{i}}} \right)} \right)^2$ to utilize the prediction from existing unsupervised methods; Prior LASSO is a supervised method which aims to incorporate some prior information for variable selection with $\hat f = \mathop {{{\mathrm{argmin}}}}\limits_{f \in {\cal H}_K} \mathop {\sum }\limits_{i = 1}^m \left( {Y_i - {\mathbf{X}}_{\mathbf{i}}{\mathbf{\beta }}} \right)^2 \,+\, \gamma _A\parallel{\mathbf{\beta }}\parallel_1 + \gamma _I\mathop {\sum }\limits_{i = 1}^m \left( {\hat Y_i^u - {\mathbf{X}}_{\mathbf{i}}{\mathbf{\beta }}} \right)^2$, where $\hat Y_i^u$ is the prediction using prior information^42^. GenoNet extends it to use both *l*_1_ and *l*_2_ norms (elastic net vs. lasso); and extends the last penalty term to unlabeled data for a semi-supervised inference.

### Model training

We trained GenoNet to provide functional predictions in 127 different cell types/tissues in ENCODE and Roadmap on a genome-wide scale, using the aforementioned MPRA validated variants in three cell lines (lymphoblastoid, liver carcinoma, and erythrocytic leukemia cell lines), and comprehensive cell type/tissue-specific epigenetic features. It is worth noting that GenoNet scores are available even in cell lines where no MPRA data are currently available (it reduces to an unsupervised method in such settings).

For these three cell lines, we used those MPRA validated variants as labeled data and randomly selected 100,000 500-bp segments across the genome where each segment includes 20 variants (25 bp apart), for a total of 2 million background variants as unlabeled data. We incorporate the pre-calculated FUN-LDA score for the training using unlabeled data^18^. For tissues/cell types where labels from high-throughput functional assays are not yet available, GenoNet reduces to an unsupervised method as described in the Methods section. The features for each labeled or unlabeled variant include seven core histone modifications (H3K4me1, H3K4me3, H3K9ac, H3K27ac, H3K9me3, H3K27me3, H3K36me3) and DNase I hypersensitivity sites in 127 different cell types and tissues from ENCODE and Roadmap, resulting in a total of 1016 functional annotations (8 annotations per tissue × 127 tissues)^10,11^. For organism-level prediction, for a given position, we took the maximum of GenoNet scores at the position across 127 tissues and cell types. Based on these training data, we generate functional predictions at every position in the genome in each cell/tissue type available in ENCODE and Roadmap. These predictions are used in all subsequent analyses.

### Semi-supervised vs. supervised vs. unsupervised approach

We investigated how GenoNet, as a semi-supervised method, performs relative to alternative supervised and unsupervised methods when the number and quality of labeled variants vary. Specifically, we compared GenoNet with Elastic-net (supervised version of GenoNet, *γ*_*I*_ = 0), LASSO, ridge regression, and support vector machine regression (SVM, ${\it{\epsilon }}$-regression with radial basis). Elastic-net, LASSO, ridge regression were implemented using the “glmnet” R package, and SVM was implemented using the “e1071” R package. The tuning parameters were chosen using tenfold cross-validation. We also include the unsupervised version of GenoNet (GenoNet-U, *γ*_*I*_ = ∞) for comparison.

**Fig. 8:**
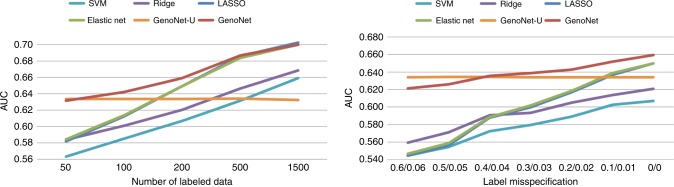
Semi-supervised vs. supervised vs. unsupervised approach. Left panel: AUROC comparison with varying number of labeled variants (*m* = 50–1500). For each replicate, *m* variants in the K562 Leukemia Cells data are randomly selected as training data in the supervised/semi-supervised methods, and the rest are used as test data. Each curve presents the average AUROC on the test data based on 1000 replicates. Right panel: AUROC comparison with various degrees of label misspecification using *m* = 200 labeled variants (high misspecification to low misspecification from left to right). GenoNet: the proposed semi-supervised method; GenoNet-U: unsupervised GenoNet, i.e. using unlabeled data only (*γ*_*I*_ = ∞); Elastic-net (supervised GenoNet), LASSO, Ridge regression were implemented using the “glmnet” R package, and SVM was implemented using the “e1071” R package. The tuning parameters were chosen using ten-fold cross-validation

We use experimental labels for 1710 MPRA validated variants for erythrocytic leukemia (K562) cell lines for the supervised methods, and 2 million genome-wide background variants with their corresponding annotations as unlabeled data. We train the semi-supervised GenoNet utilizing both labeled and unlabeled data ($\gamma _I \ge 0$), and incorporate the pre-calculated FUN-LDA predictions for the unlabeled data^18^. Overall, as we show in Fig. 8, among the supervised methods, Elastic-net and LASSO perform similarly, while ridge regression and SVM generally exhibit smaller AUC. This is likely due to the fact that only a small number of epigenetic features are expected to be predictive of MPRA labels for this cell line (K562), and therefore the variable selection conducted by Elastic-net and LASSO improves the prediction accuracy. Hence, we choose Elastic-net to be incorporated into GenoNet in the current implementation. We focus our comparisons below on the semi-supervised (GenoNet), the supervised (Elastic-net, *γ*_*I*_ = 0), and the unsupervised (GenoNet-U, *γ*_*I*_ = ∞) versions of GenoNet.

In the first scenario, we investigate the impact of the number of labeled variants. We randomly select *m* = 50–1500 variants out of 1710 variants validated by MPRA in K562 as labeled data for the training of GenoNet and the supervised methods. Then we use the trained model to predict the functional effects of the remaining 1710−*m* variants. As shown in Fig. 8, the number of labeled variants is a key factor that influences the relative performance. When the number of labeled variants is small (e.g. $m \le 100$), the supervised version (i.e. Elastic-net) has lower AUROC than the unsupervised version, GenoNet-U. This is because there are not enough labeled data to train the model well, but the unsupervised version does not require any labels. As the number of labeled variants increases from 50 to 1500, the supervised version eventually achieves a larger AUROC than the unsupervised version, as expected. The proposed semi-supervised GenoNet leads to more robust AUROC regardless of the number of labeled variants by adaptively choosing between the supervised and unsupervised versions.

In the second scenario, we investigate the impact of label misspecification. We fix the number of labeled variants to 200, and randomly set $\delta _{{\rm positive}}$ (60–0%) of true positive variants to be misspecified as control variants and $\delta _{{\rm control}}$ (6–0%) of control variants to be misspecified as positive variants. The results are described in Fig. 8. With increasing label misspecification, we observed that the AUROC for the supervised version Elastic-net decreases from 0.654 to 0.552, but of course the accuracy of the unsupervised version is not affected (AUROC of 0.634). The semi-supervised GenoNet is more robust (AUROC varies from 0.660 to 0.623) than the purely supervised version, although the AUROC is still affected by the label misspecification. Hence, as expected, the quality of labels plays a crucial role in training a model with superior prediction accuracy.

### Method evaluation under unbalanced settings

In general, for a given cell type/tissue, the proportion of functional variants in the genome is small. Since the training of GenoNet is done on fairly balanced datasets, a natural question is how the method performs in real, unbalanced settings. GenoNet is based on a logistic model for its supervised training, which is known to result in robust estimation of coefficients in an unbalanced setting where the ratio of positive and negative controls in the training data is different from that in the test data (i.e. the coefficients (except the intercept) estimated assuming prospective and retrospective designs are equivalent for logistic regression)^23^. We further investigate the issue of unbalanced test data by using the MPRA validated variants in GM12878 (LCLs) and considered a more unbalanced setting by including 19,576 additional control variants (not significant in the MPRA experiments). This results in a test dataset with 693 positive controls and 22,348 negative controls. As shown in Supplementary Figure 9, GenoNet retains its better performance in unbalanced settings, making it appropriate for predicting functional effects at genome-wide scale where the proportion of functional variants is expected to be small.

### Web-based resources

For 1000 Genomes; see http://www.1000genomes.org. For Annovar, see http://annovar.openbioinformatics.org/en/latest. For CADD, see http://cadd.gs.washington.edu.For CATO, see http://www.mauranolab.org/CATO/. For DANN, see http://jjwanglab.org/PRVCS/index.html#Download. For DeepSEA, see http://DeepSEA.princeton.edu/job/analysis/create/. For deltaSVM, see http://www.beerlab.org/deltasvm/. For Eigen, see http://www.columbia.edu/ii2135/eigen.html. For ENCODE, see https://www.encodeproject.org, https://www.encodeproject.org/data/annotations/. For Ensemble, see http://grch37.ensembl.org/index.html. For FunSeq2, see http://funseq2.gersteinlab.org. For FUN-LDA, see http://www.funlda.com. For GenoSkyline-Plus, see http://genoSkyline.med.yale.edu/GenoSkyline. For GTEx, see http://www.gtexportal.org/home. For LINSIGHT, see http://compgen.cshl.edu/~yihuang/LINSIGHT. For Roadmap Epigenomics, see http://www.roadmapepigenomics.org. For UCSC genome browser, see https://genome.ucsc.edu.

## Electronic supplementary material

Supplementary Information

## Data Availability

The data that support the findings of this study are available at the GenoNet website, http://www.funlda.com/genonet/.
