# Supplementary material for: A semi-supervised approach for predicting cell-type specific functional consequences of non-coding variation using MPRAs
Source: Nat Commun. 2018 Dec 5;9:5199. doi: 10.1038/s41467-018-07349-w (PMC6281617; doi:10.1038/s41467-018-07349-w)
Supplement: Supplementary file 1 — Supplementary Information [file 41467_2018_7349_MOESM1_ESM.pdf]

Supplementary Information for "A semi-supervised approach for predicting cell-type specific functional consequences of non-coding variation using MPRAs"

Zihuai He[1], Linxi Liu[2], Kai Wang[3], Iuliana Ionita-Laza[1,*]

[1]Department of Biostatistics, Columbia University

[2]Department of Statistics, Columbia University

[3] Raymond G. Perelman Center for Cellular and Molecular Therapeutics, Children's Hospital of Philadelphia, Philadelphia, PA 19104

[*]Correspondence to Iuliana Ionita-Laza: ii2135@cumc.columbia.edu

Supplementary Methods

Choice of tuning parameters

When we fit GenoNet, we set $\alpha = 0.5$ and $\lambda = \hat{\sigma}(Y)\sqrt{\frac{2\log p}{m}}$. When $\alpha = 0.5$, the penalty term corresponds to a combination of l_1 and l_2 norms, leading to an elastic-net model balanced between LASSO and ridge regression. In terms of prediction accuracy, LASSO works better when predictors are uncorrelated and signals are sparse while ridge regression works better when predictors are correlated and signals are more spread-out. We found that the elastic-net model with $\alpha = 0.5$ works better when some predictors are correlated and signals are sparse, which is ideal for predicting tissue-specific regulatory variants using more than a thousand cell type/tissue specific functional annotations. This is because the functional annotations are correlated and only a subset of the annotations are predictive given a target cell type/tissue.

The choice λ aims to balance the computational efficiency and prediction accuracy. By setting the tuning parameter to be data-adaptive in terms of $\hat{\sigma}(Y)$, p and m but cross-validation free, the model is feasible for future labeled data sets of larger sizes, while classical cross-validation will be very computationally intensive. For linear LASSO, under certain assumptions, $\lambda \propto \sqrt{\frac{\log p}{m}}$ has been shown to have ideal theoretical properties such as oracle property.[1,2] With stronger assumptions on the design matrix $\boldsymbol{X}$ and assuming Y has standard deviation $\hat{\sigma}(Y)$, $\lambda = \hat{\sigma}(Y)\sqrt{\frac{2\log p}{m}}$ has ideal theoretical properties with insight equivalence to Bonferroni correction.[3,4] Therefore, we used this value in GenoNet. We found that this data-adaptive λ achieves comparable performance as 10-fold cross-validation (difference in AUROC <0.02) in all datasets

evaluated in this paper, although the rigorous optimal λ is not yet available for logistic elastic net.

Comparison methods:

Methods to predict the tissue/cell type specific functional effects

- FUN-LDA - This approach is based on the latent Dirichlet allocation (LDA) model, a generative probabilistic model used often in the topic modeling literature, that allows joint modeling of data from multiple cell types and tissues. The LDA model with multiple functional classes is fit to DNase and four active histone modifications (H3K27ac, K3K9ac, H3K4me3, H3K4me1) in ENCODE and Roadmap tissues and cell types, and then for each position its posterior probability to belong to a functional class is calculated for each tissue and cell type.
- GenoSkyline - This approach is based on multivariate Bernoulli mixture models with two components. Such a model is fit to data from each tissue/cell type separately, and posterior probabilities for each variant to be in the `functional' class are calculated.
- DNase - We use negative log10 of the Poisson P-value of ChIP-seq or DNase counts relative to expected background counts.
- DeepSEA (tissue) - DeepSEA is a deep learning-based approach for predicting the epigenetic state of a sequence, including transcription factors binding, DNase I sensitivities and histone marks. It can be used to predict the chromatin effects of sequence alterations with single nucleotide sensitivity. We use DeepSEA trained on the DNase-seq data from individual cell lines as the tissue-specific scores.

- DeltaSVM - This approach is a sequence-based computational method to predict the impact on chromatin accessibility using a classifier (gkm-SVM) that encodes cell type specific regulatory sequence vocabularies. It was trained using a positive training set of putative regulatory sequences identified by DNase I hypersensitivity and a negative training set of matched negative-control sequences.

Methods to predict the organism level functional effects

- Eigen - Eigen (Eigen-PC) is an unsupervised approach for scoring variants that uses of a variety of functional annotations in both coding and noncoding regions, and combines them into one single measure of functional importance. Eigen produces estimates of predictive accuracy for each functional annotation score, and subsequently uses these estimates of accuracy to derive the aggregate functional score.

- CADD - CADD is based on a supervised approach (support vector machine) to train a discriminative model. That is, they begin with two sets of variants, one labelled as deleterious and a second one as benign, and they fit a model that best separates the two sets. Benign variants are selected by comparing the human genome to the inferred genome of the most recent shared human-chimpanzee ancestor. Alleles that are not found in the common ancestor and which are fixed in the human genome are assumed to be mostly benign. These are compared to *de novo* variants generated randomly based on models of mutation rates across the genome.

- CATO – CATO is based on training a logistic model on DNA genotyping and allelically resolved DNase-seq data in order to predict the impact of sequence variation on transcription factor occupancy. The trained model is then used to score each SNP in

dbSNP 138. It provides a recalibrated probability of affecting the binding of any transcription factor for each SNP.

- DANN - DANN uses the same feature set and training data as CADD to train a deep neural network (DNN). Unlike CADD that trains a linear kernel support vector machine to differentiate between likely benign and likely deleterious variants, and therefore cannot capture non-linear relationships among the features, DNNs can capture non-linear and interactive relationships among features.
- DeepSEA - DeepSEA is a deep learning-based approach for predicting the epigenetic state of a sequence, including transcription factors binding, DNase I sensitivities and histone marks. It can be used to predict the chromatin effects of sequence alterations with single nucleotide sensitivity. We use the functional significance score computed on the basis of DeepSEA chromatin effect predictions and evolutionary information–derived scores as the organism-level scores.
- FunSeq2 - FunSeq2 is a computational framework to annotate and prioritize somatic variants from cancer whole genome sequencing. The framework integrates large-scale genomics and cancer resources with a variant-prioritization pipeline.
- LINSIGHT - LINSIGHT is a statistical method, combining a generalized linear model for functional genomic data with a probabilistic model of molecular evolution for predicting the fitness consequences of mutations.

Prediction accuracy when using different definitions of function in the training dataset

We compared GenoNet predictions from a model trained using labels derived from MPRA validated variants, eQTLs from the Geuvadis project and TwinsUK cohort, and dsQTLs

identified from DNase hypersensitivity assays in lymphoblastoid cell lines. We used GenoNet predictions trained in one dataset to predict the functional effects for variants in other datasets. We also included the unsupervised version of GenoNet, which is robust to the definition of labels, as a benchmark method for comparison. We present the results in Supplementary Table 5. Although the prediction using exactly the same type of labels exhibits highest AUPR, AUROC and COR as expected, we observed that GenoNet trained using MPRA regulatory variants has relatively robust predictions for eQTLs and dsQTLs in terms of the AUC loss relative to when using the same type of labels for training. We currently provide predictions only using MPRA validated variants (when available), recognizing that GenoNet can be applied to other types of labels as more unbiased and comprehensive lists of experimentally validated variants from high throughput functional assays become available in the future.

Evaluation of 3C interaction variants

A series of "chromatin conformation capture" (3C) techniques have been developed in the past decade for identifying three-dimensional chromatin interactions, which are critical for a full understanding of gene regulation. We have investigated a recently reported example of 3C interaction, namely a potentially physical interaction between two SNPs rs2159100/rs12315711 residing in an enhancer and *CACNA1C* TSS in human dorsolateral prefrontal cortex, and hiPSC-derived neurons. Because of the low resolution of 3C, which of the two SNPs has a regulatory effect could not be resolved.[6] We further evaluate the two SNPs using predictions from GenoNet for ten brain regions with epigenetic data in Roadmap. We present the GenoNet predictions for these two variants in Supplementary Figure 10. We observed that both variants have relatively high GenoNet scores ranging from 0.133 to 0.435 (> 99% background variants). In addition, rs12315711 exhibits consistently higher GenoNet scores across all brain regions assessed in

Roadmap compared to rs2159100, suggesting that rs12315711 might be more likely to be a regulatory variant (see Supplementary Figure 11 for the GenoNet scores across all 127 tissues). We also investigated several organism level functional predictions for these two variants and present the results in Supplementary Table 4. The higher functional scores for Eigen, CADD, FunSeq2 and several evolutionary conservation-based metrics for rs12315711 compared to rs2159100 provide independent evidence in addition to our results based on GenoNet scores. This application illustrates the use of GenoNet score to potentially provide higher resolution interpretation of variants involved in 3C chromatin interactions.

Supplementary Table 1 GenoNet and connections to existing methods.

Parameters	Corresponding algorithms	Type
$\gamma_I \geq 0$	GenoNet	Semi-supervised
$\gamma_I = 0$	Elastic-net	Supervised
$\gamma_I = \infty$	The incorporated unsupervised method (A linear approximation)	Unsupervised

Supplementary Table 2 Cell type/tissue specific prediction of regulatory variants. For each replicate, the datasets were evenly divided into five parts: four as training data, and one as test data. Each cell presents the average AUPR (area under the precision recall curve), AUROC (area under the receiver operating characteristics curve), or COR (Pearson correlation between predicted and true labels) based on 1000 replicates. GM12878: MPRA validated variants in Lymphoblastoid Cells (693 positive variants, 2,772 control variants). HepG2: MPRA validated variants in Liver Carcinoma Cells (525 positive variants, 1,451 control variants). K562: MPRA validated variants in Erythrocytic Leukemia Cells (342 positive variants and 1,368 control variants). dsQTLs: a set of DNase I–sensitivity quantitative trait loci identified in a collection of human lymphoblastoid cell lines (559 positive variants and 2,236 control variants). GenoNet: the proposed semi-supervised algorithm.

	GM12878			HepG2			K562			dsQTLs		
	AUPR	AUROC	COR	AUPR	AUROC	COR	AUPR	AUROC	COR	AUPR	AUROC	COR
GenoNet	0.536	0.723	0.442	0.571	0.756	0.429	0.418	0.707	0.341	0.396	0.716	0.286
FUN-LDA	0.479	0.707	0.358	0.458	0.694	0.266	0.333	0.650	0.233	0.375	0.749	0.260
GenoSkyline	0.394	0.673	0.352	0.326	0.628	0.225	0.284	0.622	0.212	0.368	0.740	0.394
DNase	0.540	0.718	0.407	0.505	0.721	0.344	0.373	0.659	0.274	0.475	0.823	0.299
DANN	0.220	0.519	0.014	0.381	0.624	0.166	0.221	0.551	0.076	0.285	0.586	0.126
Eigen	0.363	0.604	0.225	0.364	0.637	0.209	0.251	0.579	0.108	0.316	0.670	0.224
Eigen-PC	0.443	0.675	0.350	0.346	0.633	0.156	0.280	0.627	0.166	0.285	0.667	0.157
CADD	0.239	0.566	0.082	0.330	0.607	0.152	0.211	0.539	0.045	0.294	0.628	0.184
FunSeq2	0.367	0.621	0.241	0.314	0.577	0.115	0.273	0.611	0.154	0.352	0.702	0.290
LINSIGHT	0.322	0.642	0.139	0.341	0.630	0.158	0.224	0.561	0.058	0.381	0.750	0.194
CATO	0.341	0.644	0.193	0.313	0.528	0.095	0.283	0.556	0.150	0.586	0.793	0.441
DeepSEA	0.387	0.690	0.222	0.399	0.668	0.188	0.280	0.605	0.097	0.599	0.821	0.361
DeepSEA (tissue)	0.278	0.539	0.144	0.204	0.688	0.281	0.200	0.580	0.177	0.346	0.591	0.215
DeltaSVM	0.311	0.593	0.178	0.407	0.672	0.264	0.314	0.585	0.192	0.591	0.751	0.477

Supplementary Table 3 Organismal level prediction of regulatory variants. Each cell presents the AUPR (area under the precision recall curve), AUROC (area under the receiver operating characteristics curve), or COR (Pearson correlation between predicted and true labels) for several organism level methods. Fine mapping eQTLs: Uniformly processed fine-mapping eQTLs for eleven studies (31,118 positive variants, 36,540 control variants); Experimentally Validated SNPs: Manually curated experimentally validated regulatory SNPs (76 positive variants, 156 control variants); Refined Causal SNPs: Refined causal SNPs in the non-coding regions from different resources including HGMD, ClinVar, OregAnno, and variants from fine-mapping candidate causal SNPs for 39 immune and non-immune diseases (5,247 positive variants, 20,988 control variants); Allelic imbalanced SNPs: Allelic imbalanced SNPs in chromatin accessibility using a large number of DNase-seq assays (8,592 positive variants, 9,678 control variants).

	Fine mapping eQTLs			Experimentally Validated SNPs		
	AUPR	AUROC	COR	AUPR	AUROC	COR
GenoNet	0.731	0.766	0.369	0.828	0.877	0.635
DANN	0.515	0.586	0.150	0.490	0.703	0.330
Eigen	0.611	0.650	0.255	0.684	0.799	0.497
Eigen-PC	0.693	0.716	0.239	0.842	0.892	0.497
CADD	0.532	0.584	0.126	0.534	0.746	0.281
FunSeq2	0.690	0.738	0.351	0.739	0.819	0.555
LINSIGHT	0.687	0.778	0.128	0.610	0.820	0.188
CATO	0.561	0.587	0.131	0.471	0.659	0.207
DeepSEA	0.614	0.670	0.288	0.672	0.779	0.414

	Refined Causal SNPs			Allelic imbalanced SNPs		
	AUPR	AUROC	COR	AUPR	AUROC	COR
GenoNet	0.549	0.811	0.458	0.896	0.921	0.612
DANN	0.246	0.585	0.119	0.567	0.635	0.238
Eigen	0.374	0.652	0.255	0.729	0.752	0.409
Eigen-PC	0.477	0.720	0.324	0.844	0.869	0.367
CADD	0.266	0.591	0.126	0.605	0.639	0.227
FunSeq2	0.485	0.742	0.397	0.788	0.827	0.472
LINSIGHT	0.427	0.781	0.161	0.814	0.881	0.236
CATO	0.307	0.614	0.147	0.863	0.835	0.444
DeepSEA	0.357	0.666	0.208	0.875	0.892	0.615

Supplementary Table 4 Functional scores for a pair of SNPs residing in an enhancer, and interacting with *CACNA1C* TSS in human dorsolateral prefrontal cortex, and hiPSC-derived neurons. The two variants cannot be distinguished by the original 3C interaction technology due to low resolution, and they are in perfect linkage disequilibrium.

SNP	rs2159100	rs12315711
Chr	12	12
Position	2346393	2346830
isTv	FALSE	TRUE
Consequence	INTRONIC	INTRONIC
priPhyloP	-0.309	-0.246
mamPhyloP	-1.104	0.394
verPhyloP	-1.275	0.389
GerpN	3.78	4.6
GerpS	-7.31	1.73
bStatistic	934	936
mutIndex	117	-102
dnaHelT	1.99	-3.14
dnaMGW	0.15	-0.21
dnaProT	-3.3	2.35
dnaRoll	1.68	-1.19
fitCons	0.054	0.054
CADD	-0.148	0.356
CADD-Phred	1.393	6.226
DANN	0.590	0.573
Eigen	-0.211	0.159
Eigen-Phred	2.260	7.302
Eigen-PC	-0.216	-0.144
Eigen-PC-Phred	0.877	5.008
LINSIGHT	0.047	0.045

Supplementary Table 5 Consistency of cell type/tissue specific predictions of regulatory variants in lymphoblastoid cell lines when the definition of labels varies. For each row, GenoNet was trained using different labels. Each cell presents the AUPR (area under the precision recall curve), AUROC (area under the receiver operating characteristics curve), or COR (Pearson correlation between predicted and true labels). The AUPR, AUROC and COR in the diagonal cells are from a random partition designed to generate independent training and test data. That is, for each replicate, the datasets were evenly divided into five parts: four as training data, and one as test data; then the average AUPR, AUROC and COR are calculated based on 1,000 replicates. MPRA: Massively parallel reporter assay (MPRA) validated variants in Lymphoblastoid Cells (693 positive variants, 2,772 control variants). Geuvadis+TwinsUK eQTLs: eQTLs in lymphoblastoid cell lines from the Geuvadis and TwinsUK studies (17530 positive variants, 36540 control variants). We further considered eQTLs with posterior probability to be causal (Brown et al.) > 0.5 (3268 positive variants, 13072 control variants). eQTLs GTEx: eQTLs in lymphoblastoid cell lines from GTEx (5684 positive variants, 22736 control variants). dsQTLs: dsQTLs in lymphoblastoid cell lines (559 positive variants, 2,236 control variants). The best performing method is bolded.

	MPRA			Geuvadis+TwinsUK eQTLs (P>0.5)			Geuvadis+TwinsUK eQTLs		
Training set	AUPR	AUROC	COR	AUPR	AUROC	COR	AUPR	AUROC	COR
Unsupervised	0.506	0.704	0.437	0.622	0.716	0.469	0.594	0.649	0.313
MPRA	0.525	0.747	0.433	0.596	0.780	0.432	0.572	0.695	0.299
Geuvadis+TwinsUK eQTLs (P>0.5)	0.342	0.652	0.278	0.781	0.917	0.677	0.775	0.874	0.553
Geuvadis+TwinsUK eQTLs	0.257	0.626	0.190	0.748	0.920	0.673	0.779	0.886	0.623
GTEx eQTLs	0.263	0.614	0.156	0.730	0.907	0.601	0.740	0.868	0.511
dsQTLs	0.482	0.668	0.396	0.535	0.645	0.383	0.593	0.667	0.275
dsQTLs_eQTLs	0.488	0.674	0.420	0.732	0.886	0.467	0.716	0.829	0.334

	GTEx eQTLs			dsQTLs			dsQTLs that are eQTLs		
Training set	AUPR	AUROC	COR	AUPR	AUROC	COR	AUPR	AUROC	COR
Unsupervised	0.366	0.641	0.201	0.334	0.668	0.211	0.500	0.736	0.363
MPRA	0.328	0.661	0.178	0.396	0.716	0.286	0.546	0.666	0.383
Geuvadis+TwinsUK eQTLs (P>0.5)	0.416	0.747	0.309	0.232	0.553	0.078	0.391	0.676	0.256
Geuvadis+TwinsUK eQTLs	0.410	0.760	0.345	0.208	0.575	0.067	0.308	0.702	0.259
GTEx eQTLs	0.447	0.775	0.356	0.220	0.535	0.051	0.358	0.662	0.202
dsQTLs	0.295	0.538	0.128	0.418	0.765	0.293	0.574	0.767	0.429
dsQTLs_eQTLs	0.374	0.719	0.161	0.371	0.726	0.241	0.543	0.744	0.383

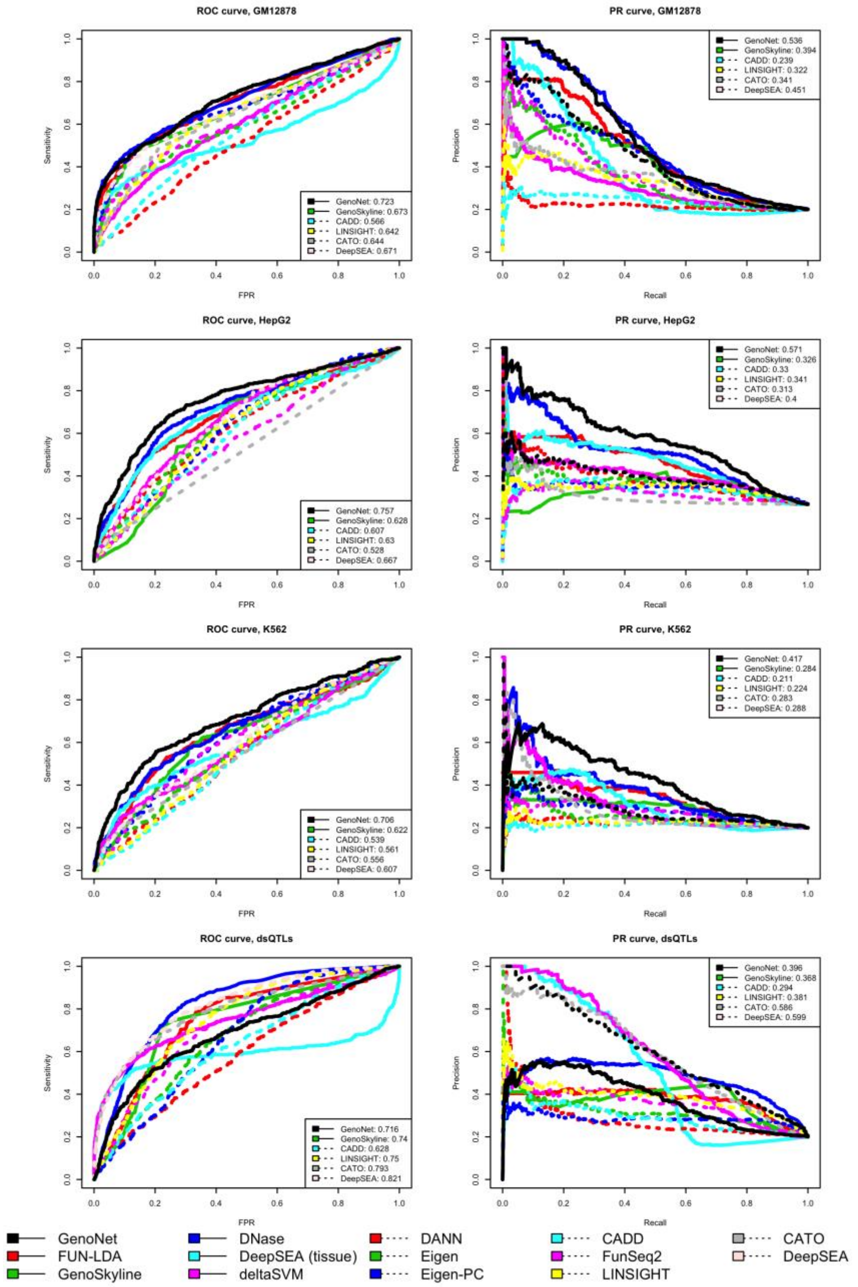

Supplementary Figure 1 ROC and PR curves for **cell type/tissue specific prediction** of regulatory variants. The figure legend presents precise AUROC/AUPR of selected scores that represent tissue specific predictions (GenoNet and GenoSkyline) and scores that represent organism level predictions (CADD, LINSIGHT, CATO and DeepSEA). Solid lines correspond to cell type/tissue specific prediction methods, and broken lines to organism level methods.

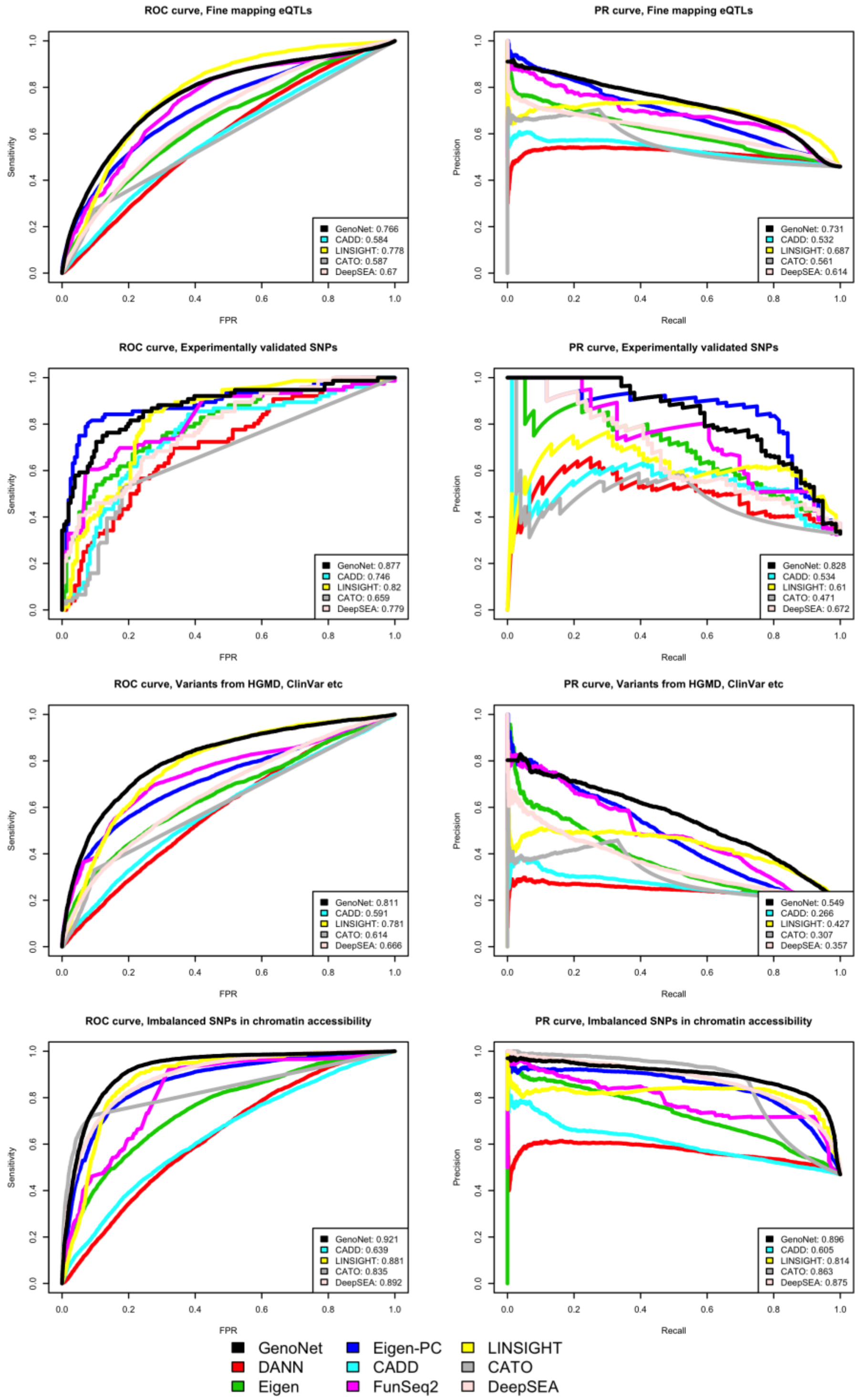

Supplementary Figure 2 ROC and PR curves for **organismal level prediction** of regulatory variants. The figure legend presents precise AUROC/AUPR of selected scores that represent tissue specific predictions (GenoNet) and scores that represent organism level predictions (CADD, LINSIGHT, CATO and DeepSEA).

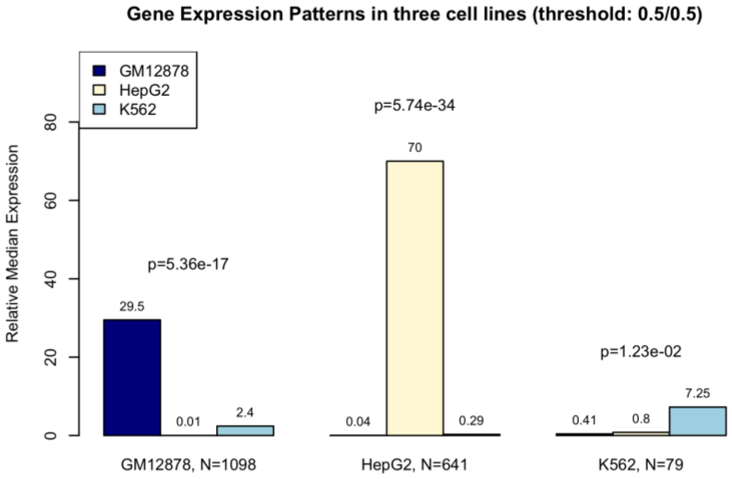

Supplementary Figure 3 Gene expression patterns for genes with cell-line specific functional variants in promoters. Each bar presents the median expression in one cell line for genes with cell line specific functional variants in promoters relative to the median expression for these genes in the other two cell lines combined. The p-values are calculated by testing whether the expression in the matched cell line is greater than the other two cell lines combined, using the Wilcoxon Rank Sum Test. The GenoNet threshold to define functional/non-functional variants in promoter region of a gene is 0.5/0.5.

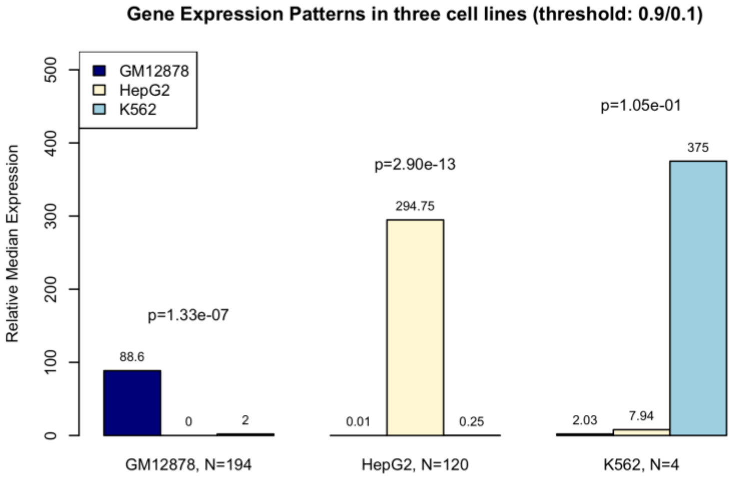

Supplementary Figure 4 Gene expression patterns for genes with cell-line specific functional variants in promoters. Each bar presents the median expression in one cell line for genes with cell line specific functional variants in promoters relative to the median expression for these genes in the other two cell lines combined. The p-values are calculated by testing whether the expression in the matched cell line is greater than the other two cell lines combined, using the Wilcoxon Rank Sum Test. The GenoNet threshold to define functional/non-functional variants in promoter region of a gene is 0.9/0.1.

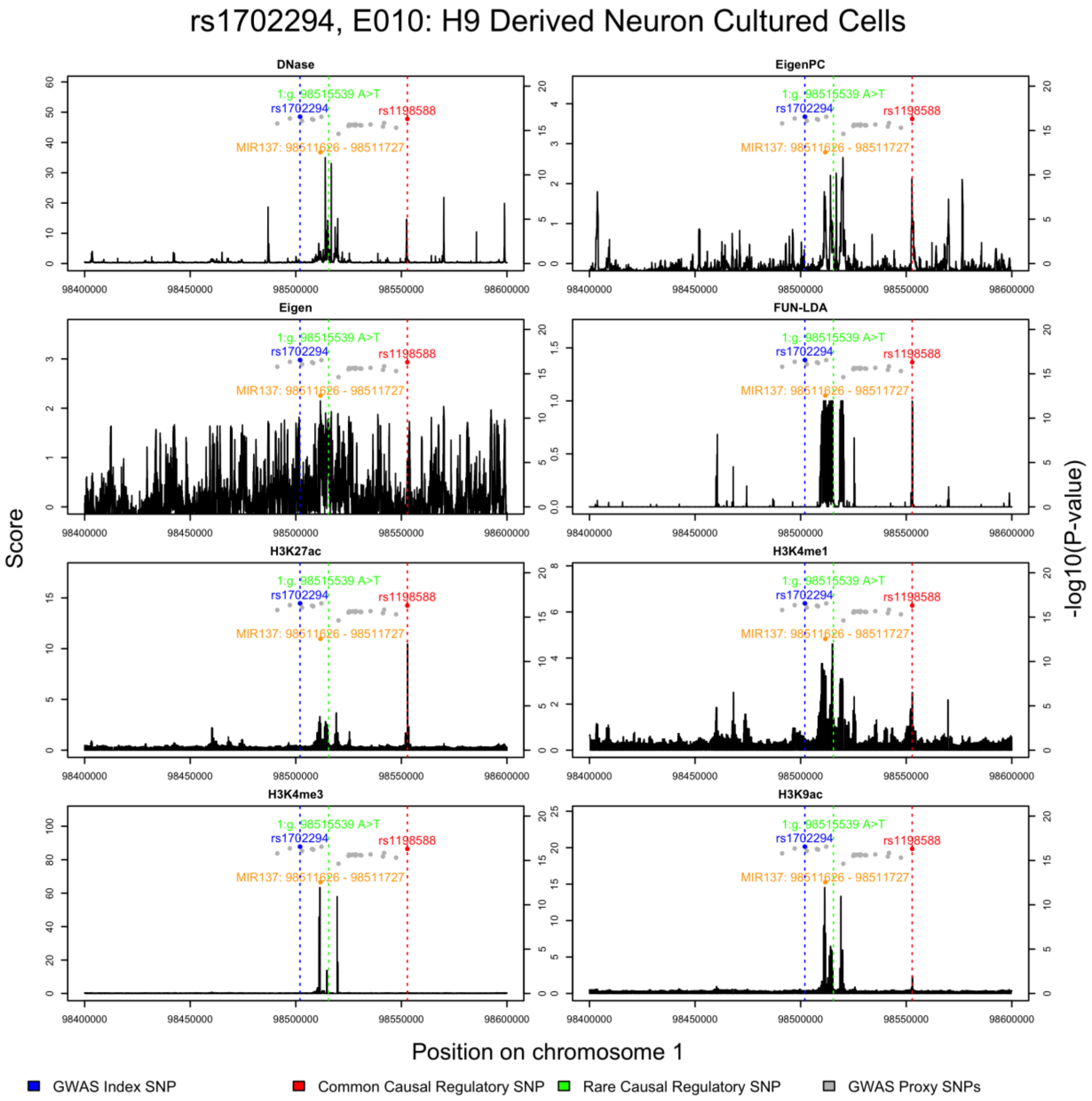

Supplementary Figure 5 Fine-mapping of the *MIR137* schizophrenia locus. Several organism level and tissue specific scores are shown.

(a) *MIR137* GWAS locus

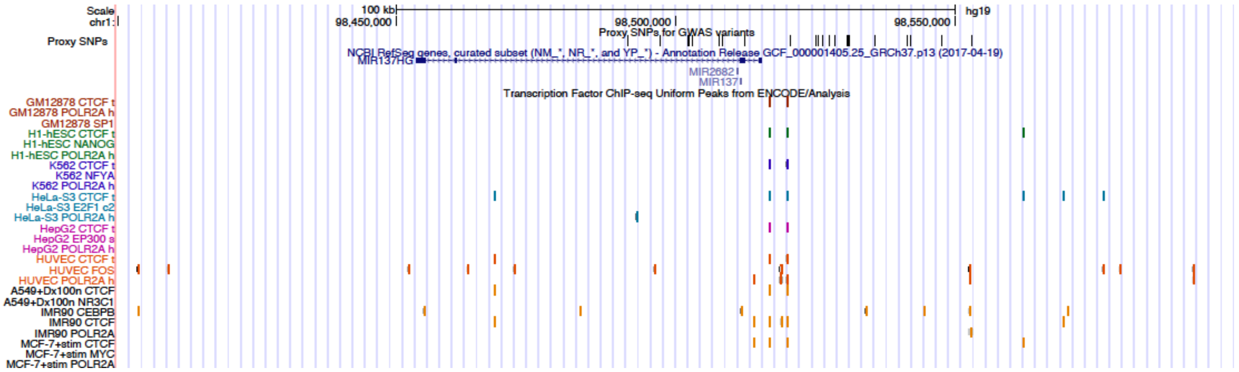

(b) 1 kb region centered at the CRISPR/Cas-9 validated functional SNP

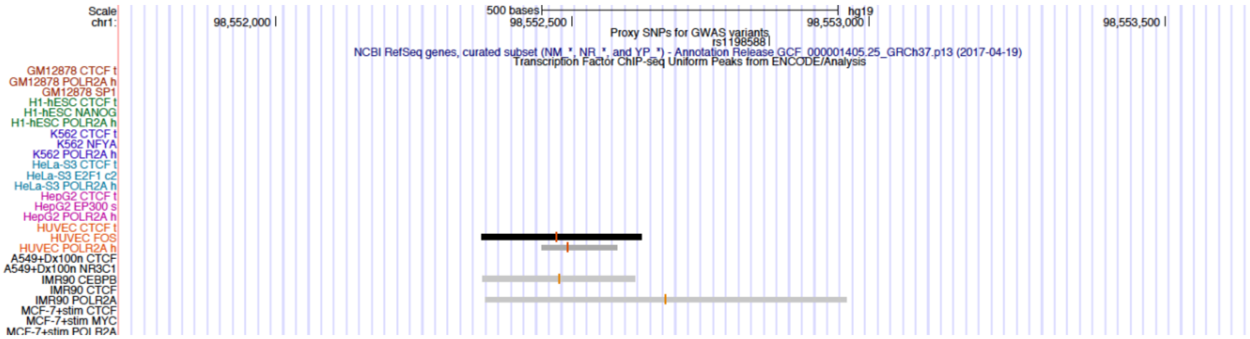

Supplementary Figure 6 Transcription factor binding sites from ChIP-seq experiments in ENCODE for the *MIR137* locus.

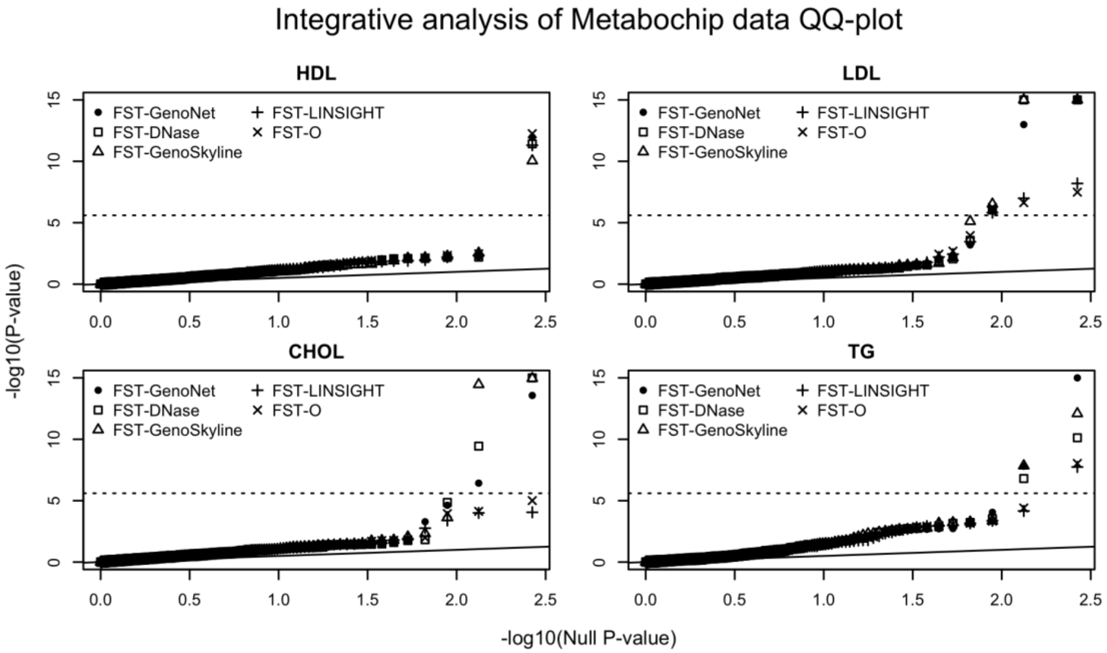

Supplementary Figure 7 QQ-plots for the meta-analyses of rare variants in the Metabochip data. The figure presents p-values for 266 genes in the 99 gold fine-mapping regions. FST-GenoNet: the functional score test proposed by He et al. 2017, integrating the GenoNet scores over 127 Roadmap tissues. FST-O: the original test combining burden and dispersion tests without integrating any functional scores (equivalent to SKAT-O). The dashed line corresponds to the gene-based genome-wide significance level ($\mathbf{2.5 \times 10^{-6}}$).

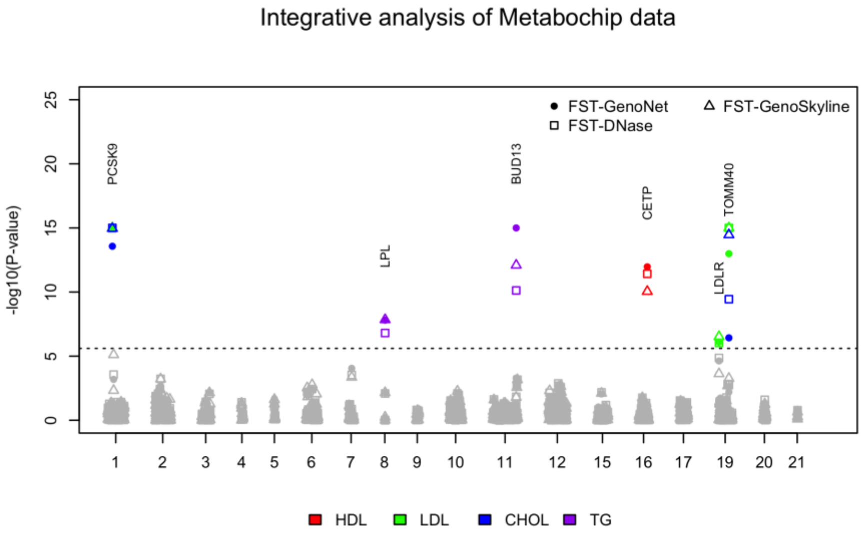

Supplementary Figure 8 Application of GenoNet scores to the meta-analysis of rare variants in Metabochip data: comparison with other tissue specific functional scores. The figure shows the p-values of 266 genes in the 99 gold fine-mapped regions in the Metabochip study. FST-GenoNet: the functional score test (FST) proposed by He et al. 2017, integrating the GenoNet scores over 127 Roadmap tissues. FST-DNase/GenoSkyline: FST integrating the DNase/GenoSkyline score. The dashed line presents the gene-based genome-wide significance level (2.5×10^{-6}). When either FST-GenoNet, FST-DNase or FST-GenoSkyline passes the significance level, all methods are highlighted for that gene-trait association. The x-axis presents the chromosome on which the genes reside.

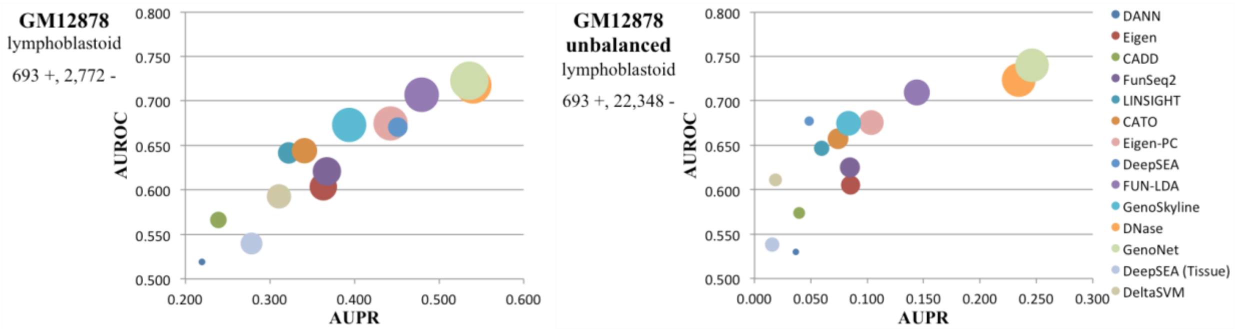

Supplementary Figure 9 Cell type/tissue specific prediction of regulatory variants with an unbalanced test data. X axis presents AUPR; Y axis presents AUROC; the bubble size represents COR. GM12878: MPRA validated variants in Lymphoblastoid Cells (693 positive variants, 22,348 control variants). GenoNet: the proposed semi-supervised algorithm.

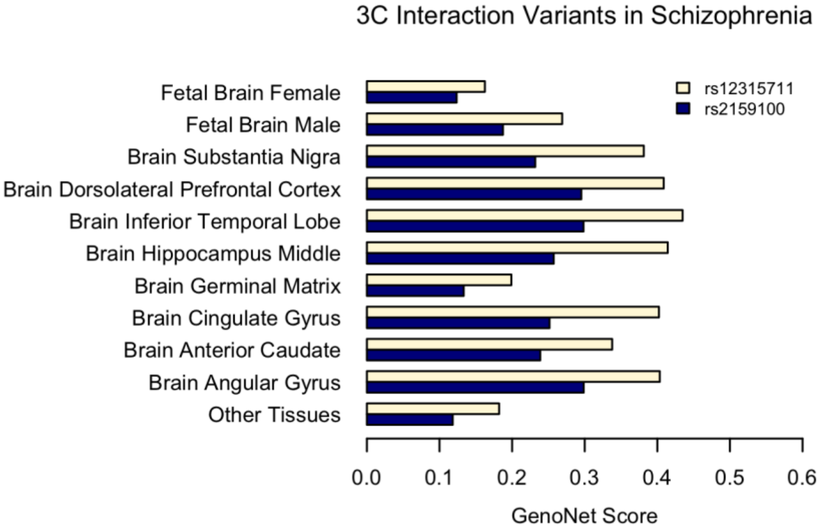

Supplementary Figure 10 Functional predictions for a pair of SNPs residing in an enhancer, and interacting with *CACNA1C* TSS in human dorsolateral prefrontal cortex, and hiPSC-derived neurons. The two variants cannot be distinguished by the original 3C interaction technology due to low resolution, and they are in perfect linkage disequilibrium. GenoNet predictions are shown for the ten brain regions in Roadmap, and the median of all the other tissues is also shown for comparison.

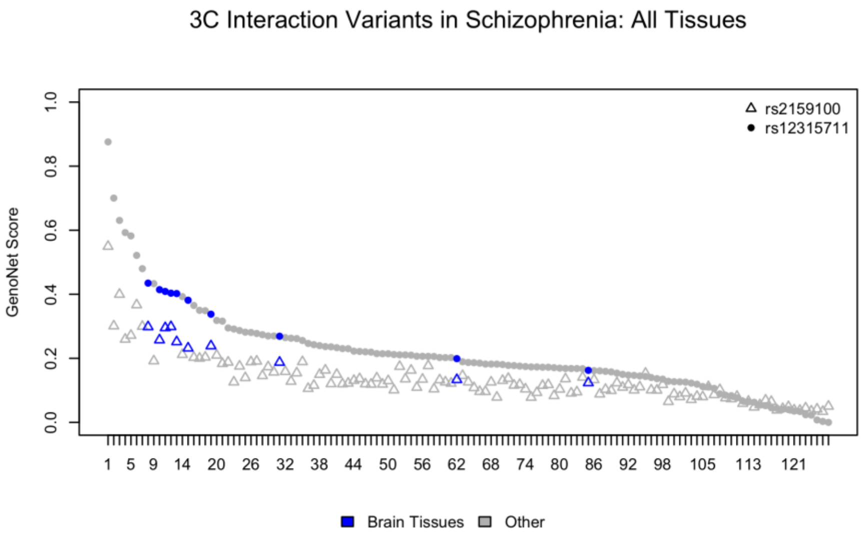

Supplementary Figure 11 Functional predictions in 127 cell types/tissues for a pair of SNPs residing in an enhancer, and interacting with *CACNA1C* TSS in human dorsolateral prefrontal cortex, and hiPSC-derived neurons. The two variants cannot be distinguished by the original 3C interaction technology due to low resolution, and they are in perfect linkage disequilibrium. The tissues are ranked by the GenoNet scores of rs12315711. The x-axis shows the tissues, and the y-axis shows the GenoNet scores. Brain tissues are highlighted in blue.

Supplementary References

1. Bunea, F., Tsybakov, A. and Wegkamp, M. Sparsity oracle inequalities for the Lasso. *Electronic Journal of Statistics*, 1, 169-194 (2007).

2. Van de Geer, S., Bühlmann, P., Ritov, Y.A. and Dezeure, R. On asymptotically optimal confidence regions and tests for high-dimensional models. *The Annals of Statistics,* **42(3),** 1166-1202 (2014).

3. Donoho, D.L. and Johnstone, J.M. Ideal spatial adaptation by wavelet shrinkage. *Biometrika*, **81(3),** 425-455 (1994).

4. Foster, D.P. and George, E.I. The risk inflation criterion for multiple regression. *The Annals of Statistics*, 1947-1975 (1994).

5. Huang, H., Fang, M., Jostins, L., Mirkov, M.U., Boucher, G., Anderson, C.A., Andersen, V., Cleynen, I., Cortes, A., Crins, F. and D'Amato, M. Fine-mapping inflammatory bowel disease loci to single-variant resolution. *Nature,* **547(7662),** 173-178 (2017).

6. Roussos, P., Mitchell, A.C., Voloudakis, G., Fullard, J.F., Pothula, V.M., Tsang, J., Stahl, E.A., Georgakopoulos, A., Ruderfer, D.M., Charney, A. and Okada, Y. A role for noncoding variation in schizophrenia. *Cell Reports*, **9(4),** 1417-1429 (2014).
